# Dual suppression of *Glossina pallidipes* using entomopathogenic fungal-based biopesticides and sterile insect technique

**DOI:** 10.3389/fmicb.2024.1472324

**Published:** 2024-12-09

**Authors:** Fidelis L. O. Ombura, Adly M.M Abd-Alla, Komivi S. Akutse, Steven Runo, Paul O. Mireji, Rosemary Bateta, Joseck E. Otiwi, Inusa J. Ajene, Fathiya M. Khamis

**Affiliations:** ^1^International Centre of Insect Physiology and Ecology (icipe), Nairobi, Kenya; ^2^Department of Biochemistry, Microbiology and Biotechnology, Kenyatta University, Nairobi, Kenya; ^3^Insect Pest Control Laboratory, Joint FAO/IAEA Centre of Nuclear Techniques in Food and Agriculture, International Atomic Energy Agency, Vienna, Austria; ^4^Unit of Environmental Sciences and Management, North-West University, Potchefstroom, South Africa; ^5^Biotechnology Research Institute, Kenya Agricultural and Livestock Research Organization, Kikuyu, Kenya; ^6^Centre for Geographic Medicine Research Coast, Kenya Medical Research Institute, Kilifi, Kenya; ^7^Department of Zoology and Entomology, University of Pretoria, Pretoria, South Africa

**Keywords:** African animal trypanosomiasis (AAT), area-wide integrated pest management (AW-IPM), *Beauveria bassiana*, *Glossina pallidipes*, human African trypanosomiasis (HAT), *Metarhizium anisopliae*

## Abstract

Tsetse flies and trypanosomosis significantly impact bovine production and human health in sub-Saharan Africa, exacerbating underdevelopment, malnutrition, and poverty. Despite various control strategies, long-term success has been limited. This study evaluates the combined use of entomopathogenic fungi (EPF) and the sterile insect technique (SIT) to combat tsetse flies. Eleven EPF isolates were tested against teneral males of *Glossina pallidipes*, focusing on mortality rates, radial growth, and impacts on fly fitness. Temperature effects on conidial growth, sporulation, and spore yield of SIT-compatible/tolerant strains were also assessed. The fungal isolates significantly influenced mortality rates in both unirradiated and irradiated (SIT-treated) males (*p* < 0.0001). *Metarhizium anisopliae* strains ICIPE 20, ICIPE 32, ICIPE 41, ICIPE 62, ICIPE 78, and *Beauveria bassiana* ICIPE 603 showed higher SIT compatibility/tolerance with LT_50_ values of 11–30 days, compared to other more virulent isolates with LT_50_ values of 4–9 days. Temperature significantly affected the radial growth of SIT-compatible EPF strains (*p* < 0.0001), with *M. anisopliae* ICIPE 78 exhibiting the fastest conidia growth at 25°C. Spore yield varied significantly across temperatures (15–40°C), and the thermal range for conidia germination of SIT-compatible strains was 8.1–45.4°C, with an optimal range of 26.7–31.1°C. Moreover, infected unirradiated females and irradiated males (donors) successfully transmitted conidia to untreated flies (receivers) without significant differences in survival rates (*p* = 0.6438) and no observed sex dimorphism. Our findings highlight the potential of combining EPF and SIT as a novel dual approach that could effectively and synergistically suppress tsetse fly populations.

## 1 Introduction

The Kinetoplastida protozoan parasites of the genus *Trypanosoma* (*Trypanosoma vivax*, *T. congolense*, *T. brucei*, *T. simiae*, and *T. suis*) are the causative agents of the lethal African Animal Trypanosomosis (AAT; nagana) and Human African Trypanosomiasis (HAT; sleeping sickness) and are cyclically transmitted by tsetse flies (Diptera: Glossinidae) (Büscher et al., 2017; Cecchi et al., 2015; Okello et al., 2022). Tsetse flies are endemic across 37 African nations (Okello et al., 2022), covering an expansive area of 10 million Km^2^ (Asfaw et al., 2022) and are associated with an estimated USD 1.55 billion in economic losses (Bitew et al., 2011; Nthiwa et al., 2015). If left untreated, HAT is typically fatal; patients gradually deteriorate, leading to coma, severe organ failure, and ultimately death (Franco et al., 2014; Jamonneau et al., 2012). Similarly, animals infected with AAT suffer significant health declines, including weight loss, reduced productivity, decreased milk production, and frequent abortions (Asfaw et al., 2022). As such, the incidence and distribution of nagana and their vectors in the afflicted areas reduce bovine productivity, whereby only 10 million out of the 165 million cattle present in Africa can be bred, and the 10 million happen to be the lowest-producing breeds (Oluwafemi et al., 2007; Nthiwa et al., 2015). These devastating impacts on both humans and animals highlight the critical importance of timely diagnosis and treatment.

Hence, tsetse flies and trypanosomiasis are consequential barriers to cattle production in sub-Saharan Africa (SSA), contributing to the undercapitalisation of land’s full potential for food production which is essential for the ever-growing African population (Morrison and MacLeod, 2011); and thus, this adds to underdevelopment, malnutrition, and poverty in the afflicted areas (Cecchi et al., 2008). Bitew et al. (2011) reported that approximately 7 million Km^2^ of the 10 million Km^2^ of the tsetse-affected area (across 37 African nations) would be suitable for mixed agriculture if AAT and HAT were eradicated by effective tsetse management. The situation is further exacerbated by large portions of SSA being the aboriginal home of AAT and its vectors, and where AAT distribution is similar to that of the biological vectors (Bardosh et al., 2013; Beadell et al., 2010; Cecchi et al., 2015).

In the recent past, several management techniques have been used to suppress tsetse flies and these include ground and aerial spraying of chemical pesiticides, the use of traps, and bush clearing (Aksoy et al., 2017). However, due to the high cost associated with these strategies, coupled with the negative environmental effects of synthetic pesticides, and highly toxic drugs, management of the tsetse has not been fully effective and become more challenging (Aksoy et al., 2017; Asfaw et al., 2022; Gooding and Krafsur, 2005). Consequently, other novel methods are being deployed such as the sterile insect technique (SIT) (Vreysen et al., 2000) and the incorporation of entomopathogenic fungi (EPF) (Maniania, 1994, 1998, 2002; Maniania et al., 2006, 2013; Wamiti et al., 2018) in area-wide integrated pest management (AW-IPM) of tsetse flies (Kabayo, 2002).

However, there is a strong knowledge gap in establishing or exploring the interactions between SIT and EPF to sustainably manage the pest. This study, therefore, aims to assess the interactions between EPF and SIT for their potential integration into a sustainable tsetse management program. The sterile insect technique is an area-wide insect pest management method where the insect pest is controlled or eradicated by affecting its reproductive capacity (Dyck et al., 2021). The technique relies on the mass-rearing of the target insect, sterilization of one sex (for instance males in tsetse fly) or both sexes (e.g., screwworm), and their release in large numbers in the natural habitat to outnumber the wild pest population (Knipling, 1955, 1959). In the case of tsetse fly, the sterile males compete with fertile males to mate with the virgin females, but the females that mate with a sterile male produce no offspring, thus reducing the next generation’s population. In addition, the sterile insects are not self-replicating and therefore, cannot become established in the environment after their release (International Atomic Energy Agency (IAEA), 2024). On the other hand, EPF are microbes that specifically infect and often kill insects and other arthropods (Molnar et al., 2010). Most are nonpathogenic to plants and relatively non-toxic to humans and animals. These EPF are crucial natural regulators of insect populations and have the potential to be used as biocontrol agents against diverse insect pests in agriculture and health sectors (Akutse et al., 2020).

Entomopathogenic fugal-based biopesticides are host-specific, eco-friendly, are persistent in the host population after introduction, are not harmful to humans and have ease of dispersal through autodissemination or horizontal transfer. Moreover, previous studies have reported the efficacy of some EPF such as *Metarhizium anisopliae*, *Beauveria bassiana*, *Isaria* sp. in controlling a plethora and diverse insect pests’ species (Akutse et al., 2020). For instance, *M. anisopliae* has been reported to be pathogenic to insects belonging to different orders including red palm weevil *Rhynchophorus ferrugineus* (Coleoptera: Curculionidae) (Gindin et al., 2006), grasshopper (Orthoptera: Acrididae) (Thomas et al., 1997), termites (Blattodea: Termitidae) (Rath, 2000), locusts *Schistocerca gregaria* (Orthoptera: Acrididae) (Arthurs and Thomas, 2001), spittlebug *Mahanarva posticata* (Homoptera: Cercopidae) (Miller et al., 2004), broad mite *Polyphagotarsonemus latus* (Acari: Tarsonemidae) (Nugroho and bin Ibrahim, 2007), *Ocinara* var*ians* (Lepidoptera: Bombycidae) (Hussain et al., 2009), spider mite *Tetranychus urticae* (Acari: Tetranychidae) (Bugeme et al., 2009), black citrus aphid *Toxoptera citricidus* (Hemiptera: Aphididae), housefly *Musca domestica* (Diptera: Muscidae) (Sharififard et al., 2011) and tsetse fly *Glossina* spp. (Diptera: Glossinidae) Maniania (1994, 1998). Recently, it was demonstrated that infection of the vector *Glossina fuscipes fuscipes* by the entomopathogenic fungus *M. anisopliae* negatively affected the multiplication of the parasite *Trypanosoma congolense* in the fly and reduced the vectorial capacity to acquire or transmit the parasite (Wamiti et al., 2018). There is therefore a significant prospect for exploring these EPF in the control of other tsetse fly species such as *Glossina pallidipes*. In addition, it is important to explore not only the potential of these EPF in tsetse fly management but also assess the interactions between tsetse-SIT and EPF to sustainably suppress the pest population. The desired EPF should be SIT-compatible by not negatively impacting the sterile males, thus allowing them to remain competitive during mating. This synergy will enhance the effectiveness and sustainability of this dual-pronged approach in tsetse population management.

Combining SIT with EPF in AW-IPM offers a robust and sustainable pest control strategy. The SIT is a highly species-specific, highly controlled and environmentally friendly technique as it does not involve introducing self-sustaining populations into the ecosystem but, has high operational costs (International Atomic Energy Agency (IAEA), 2024; Klassen and Vreysen, 2021). Entomopathogenic fungi, on the other hand, provide broad-spectrum control and are eco-friendly. However, EPF’s effectiveness can be influenced by biotic and abiotic factors, and they generally have a slower kill rate (de Faria and Wraight, 2007; Meyling and Eilenberg, 2007). Consequently, through conidia horizontal transfer (Lacey and Kaya, 2007), the sterilised males can serve as conidia donors to their conspecific mates, which can transfer the acquired spores to other mates. Therefore, integrating these two methods leverages SIT’s precision and EPF’s broad applicability, enhancing initial pest suppression and reducing operational costs while ensuring continuous pest management across varying conditions (Hajek and St Leger, 1994; Hendrichs et al., 2021). This synergistic approach underscores the critical importance of combining multiple techniques for effective and sustainable pest control.

Therefore, this study sought to (i) screen some selected EPF isolates against both non-SIT and SIT *G. pallidipes*, (ii) assess the most potent isolates with medium lethal time on the *G. pallidipes*-SIT compatible/tolerant, (iii) evaluate the relationship between temperature and radial growth of the potent SIT-compatible EPF isolates, and (iv) assess conidia acquisition, retention and horizontal conidia transfer of the potent SIT-compatible EPF isolates, as well as their effects on survival rates of *G. pallidipes*. The implications of the findings in the suppression of *G. pallidipes* in Africa are also discussed.

## 2 Materials and methods

### 2.1 Insects

Tsetse flies, *Glossina pallidipes* used in this study were obtained from the Biotechnology Research Institute (BioRI-KALRO). They were fed on alternating days on whole defibrinated bovine blood (collected from Farmers Choice Ltd., Nairobi, Kenya (S 01° 11.104′ E 036° 54.489′)). The blood was subjected to 1.0 kGy gamma irradiation (in a Gammacell 220 Excel irradiator (MDS Nordion, Ontario, Canada)) to ensure its sterility and then stored in 20 mL batches at −20°C (long-term storage). Before use, each frozen batch was thawed at 4°C then warmed to 37°C and presented to the flies (in an *in vitro* feeding method) through silicon rubber membranes (Langley, 1989).

The original *G. pallidipes* colony was derived from field collections between the Kenya-Uganda border near Tororo (Ciosi et al., 2014). The rearing was done at the Animal Rearing and Containment Unit (ARCU) at the International Centre of Insect Physiology and Ecology (*icipe*), under controlled conditions of 25 ± 2°C, 70% relative humidity (RH), and a photoperiod of 12 h light (L):12 h dark (D).

### 2.2 Irradiation of *Glossina pallidipes* males

*Glossina pallidipes* pupae were maintained at 26.5°C until emergence. Six days following eclosion, two groups of males were selected: one control group (unirradiated) and the other group designated for SIT treatment (irradiated).

The six-day-old male flies that had been fed twice were irradiated in a Gammacell 220 Excel irradiator (MDS Nordion, Ontario, Canada). The central dose rate was calibrated using transfer alanine-electron spin resonance (ESR) dosimetry against a secondary standard with a 95% CI ± 2.7%. Dose distribution mapping was performed using Gafchromic HD810 film (95% CI ± 4%). After chilling at ≈4°C, the males were placed in a 110 × 50 mm cage for irradiation with 120 Gy in air (108–132 Gy) (Langley et al., 1974). The irradiated flies were exposed to 4°C for 6 h at RH 32–34.5% in a temperature-controlled incubator, and the unirradiated control group remained in the colony-holding conditions. Upon removal from the low-temperature chamber, the flies were immediately returned to colony-holding conditions in standard colony cages (18 cm x 7 cm x 4.5 cm) overnight for acclimatization before the bioassays. Any overnight mortality among the experimental flies in the colony cages was recorded. Thirty (*n* = 30) males of *G. pallidipes* were used per treatment and replicated three times for the bioassays.

### 2.3 Fungal cultures

For this study, 10 *Metarhizium anisopliae* strains (ICIPE 7, ICIPE 18, ICIPE 20, ICIPE 30, ICIPE 32, ICIPE 40, ICIPE 41, ICIPE 62, ICIPE 69, ICIPE 78) and one *Beauveria bassiana* strain ICIPE 603 were obtained from the International Centre for Insect Physiology and Ecology (*icipe*)’s germplasm maintained by the Arthropod Pathology Unit and were screened for their efficacy against tenerals of *G. pallidipes*. The isolates were sub-cultured on Sabouraud Dextrose Agar (SDA, Oxoid Ltd., Hampshire, United Kingdom) except *B. bassiana,* which was sub-cultured on Potato Dextrose Agar (PDA, Oxoid Ltd) and maintained at 25 ± 2°C in complete darkness until sporulation. Darkness is essential for the EPF’s consistent growth conditions (Pitt and Hocking, 2009) and enhanced sporulation (Inglis et al., 2001). The selected strains of *M. anisopliae* and *B. bassiana* do not produce resting spores in the traditional sense, such as zygospores in Zygomycota or chlamydospores in certain other fungi. Instead, they primarily form conidia (asexual spores), which are the main propagules used for infection and dissemination (St Leger, 1995). Conidia were harvested from three 3-week-old sporulated plates using sterile spatulas. The harvested conidia were mixed in 10 mL of sterile distilled water containing 0.05% (w/v) Triton X-100 (Sigma Aldrich, Inc., Missouri, United States) in a 30 mL universal bottle containing glass beads (5–10 beads of 3 mm in diameter per bottle) and vortexed for 5 min at about 700 rpm (using a ZX3 Advanced Vortex Mixer, VELP Scientifica Srl, Usmate, Italy) to produce homogenous conidial suspensions. Conidial concentrations were then determined using a new improved Neubauer chamber (Paul Marienfeld GmbH & Co. KG, Lauda-Königshofen, Germany) under the Leica DM2000 LED light microscope (Leica Microsystems (UK) Ltd). Prior to every bioassay, the conidia viability of each isolate was tested; where the conidial suspensions were then adjusted to a concentration of 3 × 10^6^ conidia mL^−1^ then 0.1 mL of the suspension evenly spread on SDA or PDA plates. Plates were sealed with parafilm and incubated at 25 ± 2°C in complete darkness for 18 h. Each plate represented a replicate, and four replicates were done for each isolate and the treatments were arranged in a completely randomized design (CRD). After 18 h post-incubation, the plates were unsealed, fixed with a drop of fixative lactophenol cotton blue, and randomly covered with four sterile microscope coverslips (2 × 2 cm). Percentage conidial germination was determined from 100 randomly selected conidia on the surface covered by each coverslip under a Leica DM2000 LED light microscope (×40). Conidia were considered germinated when the length of the germ tube was at least twice the diameter of the conidium (Goettel and Inglis, 1997; Maniania, 1992). The germination rate determines whether the selected EPF isolate will be used for the bioassay. Over time, spore viability is affected by several factors; hence, it was monitored carefully. No EPF strain with ≤80% spore germination was used. If the spore germination rate fell below this threshold, the EPF strain was not used but was instead exposed to a susceptible host, then pin-isolated to regain its virulence, sub-cultured, and finally used for bioassays (Goettel and Inglis, 1997).

### 2.4 Fungal mass-production

Due to the large quantities of fungi needed for the bioassays, mass production of the EPF isolates was done on rice substratum (separately for each isolate) in 600 mm × 350 mm Breathable Bio Control PP Bags (24 × 14) with double B filter (Unicorn Imp. & Mfg. Corp, Plano, Texas, US). The rice (2 Kg in each bag) was autoclaved for 1 h at 121°C and 15 psi, then allowed to cool in a Purifier Logic+ Class II, Type A2 Biosafety Cabinet (Labconco Corp, Kansas City, MO, United States), after which inoculation was done using 50 mL 3-day old culture of blastospores of the desired fungal isolate. The blastospores were prepared by aseptically scoping pure conidia into a 250 mL Erlenmeyer flask containing 50 mL of sterile liquid broth [30 gL^−1^ glucose (Sigma Aldrich)], 30 gL^−1^ yeast extract (Oxoid Ltd), 15 gL^−1^ peptone (Oxoid Ltd) which was then incubated at 25 ± 2°C at 150 rpm for 3 days in a New Brunswick™ Innova® 44/44R incubator shaker (Eppendorf AG, Hamburg, Germany). The inoculated rice bags were incubated for 3 weeks at 25 ± 2°C and 40–70% relative humidity (RH), with continuous monitoring for quality control. After this, the rice with fungal growth was placed in basins (33 × 25 × 13 cm), to allow the conidia to dry at 25 ± 2°C, for 7 days. The conidia were harvested through agitating using MycoHarvester 6™ (VBS Ltd., United Kingdom; http://www.dropdata.net/mycoharvester/). The harvested pure conidia were packed in sterile zip-lock bags and then stored at 4 ± 2°C until used in downstream bioassays. Before any bioassays, conidia viability was assayed as described above.

### 2.5 Pathogenicity of *Metarhizium anisopliae* and *Beauveria bassiana* isolates against *Glossina pallidipes*

Experimental insects (irradiated and unirradiated *G. pallidipes*) were infected with entomopathogenic fungi in separate batches using velvet carpet fabric layered with dry conidia that covered the inside of a cylindrical plastic tube (infection chamber) (95 × 48 mm) and had white nylon netting over one end (Dimbi et al., 2003; Wamiti et al., 2018). Dry conidia (0.1 g) were spread evenly onto the velvet material. Each batch consisted of 30 males (6 days old), which were enclosed within the infection chamber and exposed to the fungus for 12 h, after which they were transferred and placed into clean fungus-free cylindrical plastic tubes (95 × 48 mm), and maintained at 25 ± 2°C and 55% RH in a MIR-554 cooled incubator (PHC Europe B.V., Nijverheidsweg, Netherlands), at a photoperiod of 12 h Light: 12 h Dark. A total of 90 tenerals were eventually used (*n* = 30, replicated three times) for each treatment. The fungal_exposed flies were fed on bovine blood as described above. The control treatments (unirradiated control groups) were not exposed to any fungal strains and the experiments were run for 15 days, where the mortality of the flies was recorded. For all mortality observed, mycosis tests were conducted to confirm whether death was due to infection by the fungus used in the bioassays. The insect cadavers were surface_sterilized with 70% alcohol, rinsed three times in distilled water, and then kept separately in 90 mm Petri dishes lined with sterile moistened filter paper to allow for fungal outgrowth and verify if mortality could be attributed to the respective fungal isolates they were treated with. Mortality due to fungal infection was confirmed by the presence of hyphae and conidia on the surface of the cadavers. The median lethal time (LT_50_) of the EPF isolates’ efficacy was calculated (15 days post fungi exposure) to further select the SIT-compatible EPF strains.

### 2.6 Radial growth rate of the selected SIT-compatible entomopathogenic fungal isolates

The colony diameter and radial growth rate of the best selected SIT-compatible EPF isolates (*Metarhizium anisopliae* isolates ICIPE 20, ICIPE 41, ICIPE 62 and ICIPE 78, and *Beauveria bassiana* ICIPE 603) were assayed under different temperatures, i.e., 15, 20, 25, 30, 35, and 40°C, respectively. To assess the effect of temperature on radial growth and colony diameter of these five selected candidates of EPF isolates, they were cultured on SDA plates except ICIPE 603 which was cultured on PDA and maintained at 25 ± 2°C under complete darkness as described above for 3 days to obtain mycelial mats. On the third day post-incubation, fresh SDA or PDA plates (9 cm diameter) were prepared. Using a marker pen, lines were drawn at the back of the plate to divide the plate into four quadrants as described by Agbessenou et al. (2021). Mycelial mats were cut from culture plates into round agar plugs using an eight-mm diameter cork borer. Each agar plug (*ca.* 5 mm thick) was then transferred onto the centre of a fresh SDA or PDA medium plate from which a similar size plug of media had been previously removed using the same cork borer. The plates with implanted mycelial plugs were sealed with Parafilm membrane and incubated in complete darkness at the defined temperatures, 15, 20, 25, 30, 35, and 40°C. Radial growth was recorded daily for 14 days using two cardinal diameters (a flexible ruler), through two orthogonal axes (the four quadrants) previously drawn on the bottom of each Petri dish to serve as a reference (Fargues et al., 1992). The experiment was replicated four times with each replicate originating from a different culture plate, where the radius of the growing fungi was measured, for all four quadrants. Fourteen days post-incubation conidia were then harvested by scraping the surface of the sporulated cultures from each plate using a sterile spatula. On the 14th day after taking the daily radius readings, the spore yield of each fungal plate was determined followed by germination assays of all the plates per temperature treatment. The harvested conidia were then suspended in 10 mL sterile distilled water containing 0.05% Triton X-100 and vortexed for 5 min at about 700 rpm (using a ZX3 Advanced Vortex Mixer, VELP Scientifica Srl, Usmate, Italy) to break conidial clumps and ensure a homogenous suspension. Conidial concentrations were quantified using an improved Neubauer hemocytometer under a light microscope as described above (Goettel and Inglis, 1997).

### 2.7 Entomopathogenic fungal isolates conidia horizontal transfer, conidia acquisition and retention by *Glossina pallidipes*

For conidia acquisition, retention and horizontal transfer bioassays, two potent *M. anisopliae* isolates ICIPE 20, and ICIPE 78 that were SIT-compatible/tolerant and responded well to the temperature from the above bioassay were used. Before evaluating whether EPF-exposed males and females could transmit conidia to their conspecific mates, we determined whether the amount of conidia carried by *G. pallidipes* individuals varied across time and between sexes. Hence, 0.1 g of dry conidia were evenly lined on a velvet carpet fabric in an infection chamber (95 × 48 mm cylindrical tube), then 10 *G. pallidipes* teneral males (conidia donors) were introduced into the infection chamber, were exposed to the fungus for 12 h, and then transferred into clean rearing cages with 10 healthy virgin fungus-free *G. pallidipes* females (conidia receivers). The conidia donors (males) were interacted with the conidia receivers (females) for 24 h then all the conidia donors were removed and kept separate (Dimbi et al., 2003; Wamiti et al., 2018). The conidia receivers were later collected at random (three female flies per day per replicate) on 1st, 3rd, and 5th day post exposure to fungus-challenged *G. pallidipes* males (conidia donors), then individually placed into universal bottles with 2 glass beads and 1 mL sterile distilled water containing 0.05% Triton X-100. The universal bottles with the EPF exposed flies were vortexed for 5 min at 700 rpm (using a ZX3 Advanced Vortex Mixer (VELP Scientifica Srl, Usmate, Italy) to release conidia from the insect’s body), then the conidia on their bodies quantified as already described above, using an improved Neubauer hemocytometer. This experiment was set in a completely randomized design, replicated three times and maintained in similar conditions as described above.

An inverse bioassay was set up whereby *G. pallidipes* females were used as conidia donors while the *G. pallidipes* teneral males were used as the conidia receivers. In this setup, 10 healthy virgin *G. pallidipes* females were contaminated with 0.1 g of dry conidia (as described above) and were later introduced into rearing cages with 10 fungus-free teneral *G. pallidipes* males (conidia receivers), after which the conidia donors (females) were removed after 24 h and the conidia receivers (males) collected at random as already described above. The conidia from the flies’ bodies were recovered and quantified as already described above. This experiment was also set in a completely randomized design and replicated three times. In all the bioassays, we considered fungus-free males and females as control treatments. Exposure of the flies to *M. anisopliae* ICIPE 20 and ICIPE 78 was done separately.

### 2.8 Effects of SIT-compatible EPF isolates on the survival of male and female *Glossina pallidipes*

Another bioassay was set up as described above in the horizontal transmission where the insects were infected using the infection chamber. Two *M. anisopliae* isolates ICIPE 20 and ICIPE 78 were used in this bioassay. This experiment was done in two setups. In the first, teneral males were conidia donors and females as conidia receivers in one scenario, while in the second, females were conidia donors and males as conidia receivers (as described in section 2.7 above). Both the donors’ and the receivers’ treatment groups (for both scenarios) were observed daily for mortality for 15 days, and mycosis test was conducted on the recorded cadavers as described above. A similar setup of bioassay was done with male and female flies which were not exposed to any fungus and used as a control treatment. This experiment was set in a completely randomized design and replicated three times. Mortality due to fungal infection was confirmed through mycosis test as described above by assessing the presence of hyphae and conidia on the surface of the cadaver. The survival rates of both the males and females in the donor and receiver groups were then calculated.

### 2.9 Temperature-dependent modelling on the radial growth of the fungi

The absolute radial growth (slope; mm/day) was determined by plotting the radial growth data against time (days) and fitted to a linear regression model (*γ* = *βx* + *c*) in Microsoft Excel (Microsoft 365), whereby γ represents the radial growth (indicating size of the mycelium at a given time), β represents the slope of the linear regression (indicates change of radial growth over time), *x* represents time (measured in days) and *c* represents the y-intercept of the linear regression line. The computed radial growth data was then fitted into both linear and nonlinear temperature-dependent models to establish the relationship between EPF growth rate and temperature. This association between the temperature regimes and the fungal growth was estimated using the linear model *y*(t) = *a* + *bt*, whereby *y* is the growth rate, *t* is the ambient temperature, and intercept (*a*) and slope (*b*) are the model parameters (Agbessenou et al., 2021). However, in extreme temperatures, the growth rate cannot be accurately captured by a linear function (Vidal et al., 1997). Hence, we selected two empirical nonlinear models (Lactin 1 and CTMI) based on their capacity to explain and predict at least three biologically significant and physically comprehensible namely minimum, optimum, and maximum thermal thresholds as well as maximal growth response of the EPF at specific temperatures (Omuse et al., 2022). The selected nonlinear models were:

Lactin 1 (Lactin et al., 1995):


(1) $$\mathrm{\mu m}=a{\left(T-\mathrm{Tmin}\right)}^{2}\left(\mathrm{Tmax}-T\right)$$

CTMI (Rosso et al., 1995):


(2) $$\mathrm{\mu m}=\frac{\mathrm{\mu opt}\left(T-\mathrm{Tmin}\right){\left(T-\mathrm{Tmin}\right)}^{2}}{\mathrm{Topt}-\mathrm{Tmin}\left[\begin{array}{l} \left(\mathrm{Topt}-\mathrm{Tmin}\right)\left(T-\mathrm{Topt}\right)-\\ \left(\mathrm{Topt}-\mathrm{Tmax}\right)\left(\mathrm{Topt}+\mathrm{Tmin}-2T\right) \end{array}\right]}$$

In these models, *μm* stands for our absolute radial growth (mm day^−1^) at a particular temperature (*T*), *Tmin* denotes the theoretical minimum temperature, *Topt* denotes the ideal temperature, and *Tmax* denotes the maximum temperature. In Equations 1, 2, the parameter *a* is an empirical constant (Lactin et al., 1995).

### 2.10 Data analysis

Data on conidial germination were analyzed using a generalized linear model (GLM), which was predicated on the assumption of a binomial distribution with the *log* link function. Using Abbott’s formula (Abbott, 1925), the cumulative per cent mortality was adjusted to factor in mortality in the controls, and their normality was tested using the Shapiro–Wilk test (Shapiro and Wilk, 1965) followed by analysis using *poisson* in a GLM for binomial distribution. Subsequently, the data underwent one-way analyses of variance (ANOVA). The *dose.p* function was utilized to analyze all time-mortality data with GLM to estimate LT_50_ estimates and the slopes of the regression curves. The resulting LT_50_ values and their corresponding slopes were further analyzed via ANOVA. For the analysis of *G. pallidipes* conidia horizontal transmission and retention data, a GLM was applied, followed by ANOVA. Tukey’s HSD *post-hoc* test was employed for pairwise comparisons to distinguish means if significant differences were observed, with alpha (*α*) set at 0.05. Results were considered significant at a 95% confidence level (*p* < 0.05).

The *survival* package (Therneau, 2015) was used to perform Kaplan–Meier survival analyses with the Mantel-Cox log-rank *chi*-squared test to evaluate the effect of SIT-compatible entomopathogenic fungal isolates on the survival rates of *G. pallidipes* (both conidia donors and receivers). Since the data were normally distributed (Shapiro–Wilk test: *p* < 0.05), they were subjected to ANOVA followed by Tukey’s HSD *post-hoc* test. Furthermore, to investigate whether insect sex influences conidia spread between the *G. pallidipes* conidia donors (males) and conidia receivers (females), we used the unpaired *t*-test to compare the median lethal time between conidia donors and conidia receivers.

For the temperature-dependent modelling of the SIT-compatible isolates, absolute radial growth was computed using a linear regression model then was fitted into both linear and nonlinear temperature-dependent models. To obtain weighted least-square estimates of the nonlinear model’s parameters, the *nls* function was employed for datasets with a non-zero residual sum of squares and the *nlsLM* function from the *minpack*.*lm* package for datasets with zero residual sum of squares (Elzhov et al., 2016). Using the *rcompanion* package, the Akaike’s Information Criterion (AIC) and adjusted *R*^2^ (adj. *R*^2^) goodness-of-fit metrics of the fitted models were compared (Mangiafico, 2020). The adj. *R*^2^ was calculated from pseudo *R*^2^ to account for the degrees of freedom in the fitted models as described by Kvålseth (1985). Hence, the best-fitting model was determined to have the least AIC and the highest adj. *R*^2^. The *nonnest2* package’s implementation of Vuong (1989) non-nested likelihood ratio test led to the statistical establishment of equally well-fitting models (Merkle and You, 2020). Unweighted means were used to present the findings of statistical estimations for the parameters. All statistical data analyses were performed using R software (v. 4.1.0; R Core Team, 2020).

## 3 Results

### 3.1 Pathogenicity of *Metarhizium anisopliae* and *Beauveria bassiana* isolates against *Glossina pallidipes*

**Figure 1:**
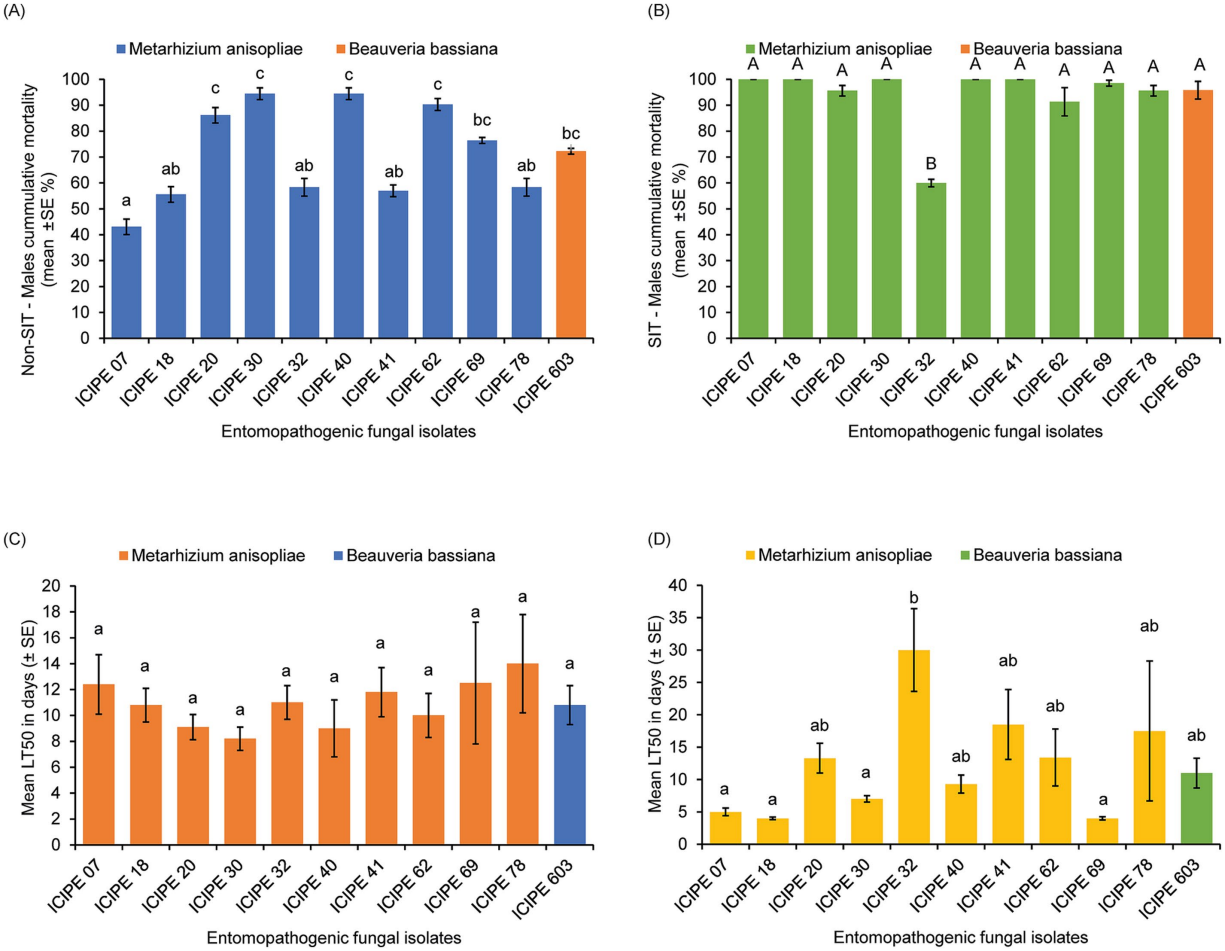
Pathogenicity and virulence of the different entomopathogenic fungal (EPF) isolates against tenerals of *Glossina pallidipes* (*n* = 30). **(A)** Cumulative mortality of EPF-challenged unirradiated *G. pallidipes* males. **(B)** Cumulative mortality of EPF-challenged irradiated *G. pallidipes* males. **(C)** Median lethal time (LT_50_) of EPF-challenged unirradiated *G. pallidipes* males. **(D)** The median lethal time (LT_50_) of EPF-challenged irradiated *G. pallidipes* males. Means were separated using Tukey’s HSD *post hoc* test. Error bars indicate the standard error of the means, and the bars/means followed by different letters are significantly different (*p* > 0.05).

The viability of the EPF isolates used in this study ranged between 82.5 ± 1.95 to 99.75 ± 0.22% after 18 h incubation with no significant difference (df = 10; *p* = 0.9554) among the isolates (Table 1; Supplementary Figure S1). All the EPF isolates screened against the unirradiated (Non-SIT) teneral males of *G. pallidipes* showed significant differences (*χ*^2^ = 6.02, df = 10, *p* < 0.0001) on the mortality of the unirradiated *G. pallidipes* males 15 days post-infection (Figure 1A), with *M. anisopliae* strains ICIPE 30, ICIPE 40, and ICIPE 62 causing the highest mortalities (94.44 ± 2.27%, 94.44 ± 2.27%, and 90.28 ± 2.27% respectively) compared to the other fungal isolates; while *M. anisopliae* ICIPE 7 caused the least mortality of 43.06 ± 3.00% (Figure 1A). In addition, there was significant variation in the mycosis rates (*χ*^2^ = 4.58, df = 10, *p* < 0.0001) among the various EPF-challenged unirradiated mycosed cadavers of *G. pallidipes,* with mycosis rates ranging between 86.11 ± 2.76 to 96.67 ± 2.27% (Supplementary Table S1).

**Table 1:** Source, identity, pathogenicity, spore viability, and virulence/lethal median times of the entomopathogenic (EPF) isolates against irradiated (SIT) *Glossina pallidipes* males.

EPF strain	Isolate	Isolation source	Location	Year of isolation	Spore viability prior to use in bioassays (% ± SE)	Mortality (% ± SE)	LT50 (Days ± SE)
*Metarhizium anisopliae*	ICIPE 07	*Rhipicephalus appendiculatus*	Rusinga Island, Kenya	1996	95.5 ± 1.15a	99.96 ± 0.04a	5 ± 0.6A
	ICIPE 18	Soil	Mbita, Kenya	1989	92.25 ± 1.24a	99.96 ± 0.04a	4 ± 0.2A
	ICIPE 20	Soil	Migori, Kenya	1989	96 ± 0.61a	95.59 ± 2.05a	13.3 ± 2.3AB
	ICIPE 30	*Busciola fusca*	Kendu Bay, Kenya	1989	82.5 ± 1.95a	100 ± 0a	7 ± 0.5A
	ICIPE 32		Australia	2003	96 ± 1.06a	59.94 ± 1.45b	30 ± 6.4B
	ICIPE 40	Soil	Kitui, Kenya	1990	96.75 ± 1.14a	99.96 ± 0.04a	9.3 ± 1.4AB
	ICIPE 41	Soil	Migori, Kenya		97.75 ± 1.14a	99.96 ± 0.04a	18.5 ± 5.4AB
	ICIPE 62	Soil	Kinshasa, DR Congo	1989	99.25 ± 0.65a	91.33 ± 5.47a	13.4 ± 4.4AB
	ICIPE 69	Soil	Kinshasa, DR Congo	1990	99.75 ± 0.22a	98.51 ± 1.17a	4 ± 0.25A
	ICIPE 78	*Temnoschoita nigroplagiata*	Ungoe, Kenya	1990	94.5 ± 1.60a	95.61 ± 2.05a	17.5 ± 10.8AB
*Beauveria bassiana*	ICIPE 603	Hymenoptera	Taita, Kenya	2007	83 ± 0.94a	95.79 ± 3.38a	11 ± 2.3AB

Values with different lowercase letters within the same column are significantly different from each other.

On the other hand, the mortality rates of the EPF-challenged SIT-treated males of *G. pallidipes* were significantly different (*χ*^2^ = 5.34, df = 10, *p* < 0.0001) among the fungal isolates (Figure 1B; Table 1), where *M. anisopliae* isolates ICIPE 30, ICIPE 07, ICIPE 18, ICIPE 20, ICIPE 40 and ICIPE 41 recorded the highest mortalities (100 ± 0%, 99.96 ± 0.04%, 99.96 ± 0.04%, 99.96 ± 0.04% and 99.96 ± 0.04% respectively); while *M. anisopliae* ICIPE 32 caused the lowest mortality of 59.94 ± 1.45% 15 days post-infection (Figure 1B). However, there was no significant difference (*χ*^2^ = 1.75, df = 10, *p* = 0.5062) in the mycosis rates of the EPF-challenged SIT-treated cadavers of *G. pallidipes* among the fungal isolates, and the mycosis rates ranged between 91.48 ± 1.42–100 ± 0.0% (Supplementary Table S1).

There was no significant difference (*χ*^2^ = 2.78, df = 10, *p* = 0.9861) in the median lethal times (LT_50_) among the various screened EPF against the unirradiated *G. pallidipes* with the LT_50_ values ranging between 8.2 ± 0.9 to 12.5 ± 4.7 days (Figure 1C). However, there was significant difference (*χ*^2^ = 47.35, df = 10, *p* < 0.0001) in the median lethal times among the various screened EPF isolates against irradiated *G. pallidipes*. *Metarhizium anisopliae* strains ICIPE 20, ICIPE 32, ICIPE 41, ICIPE 62, ICIPE 78 and *B. bassiana* ICIPE 603 recorded the highest LT_50_ values (13.3 ± 2.3, 30 ± 6.4, 18.5 ± 5.4, 13.4 ± 4.4, 17.5 ± 10.8 and 11 ± 2.3 days, respectively) and were selected as suitable candidates for compatibility/tolerance with SIT (Figure 1D; Table 1). However, *M. anisopliae* ICIPE 32 was not used in temperature-dependent assays, modelling, conidia acquisition and transfer bioassays due to the very long median lethal time it had (30 ± 6.4 days) (Table 1). This long median lethal time could be an indication of slower activity against the target pest or maybe the EPF (ICIPE 32) is less adapted to the tsetse’s environment, which may limit its ability to establish infection, or the tsetse (host) may have mechanisms that enable it to evade infection allowing the pathogen to persist within the host for an extended period. Conversely, *M. anisopliae* ICIPE 40 (despite having a high mortality rate of 99.96 ± 0.04%; Table 1) was not also used in temperature-dependent assays, modelling, conidia acquisition and transfer bioassays due to its relatively short median lethal time (9.3 ± 1.4 days) (Table 1). Moreover, Mutika and Parker (2014) demonstrated that the average longevity of sterilised and starved *G. pallidipes* males was ≥17 days hence any EPF with LT_50_ values of more than 20 days and lower than 10 days would not be suitable for SIT.

### 3.2 Effect of temperature on conidia germination

**Figure 2:**
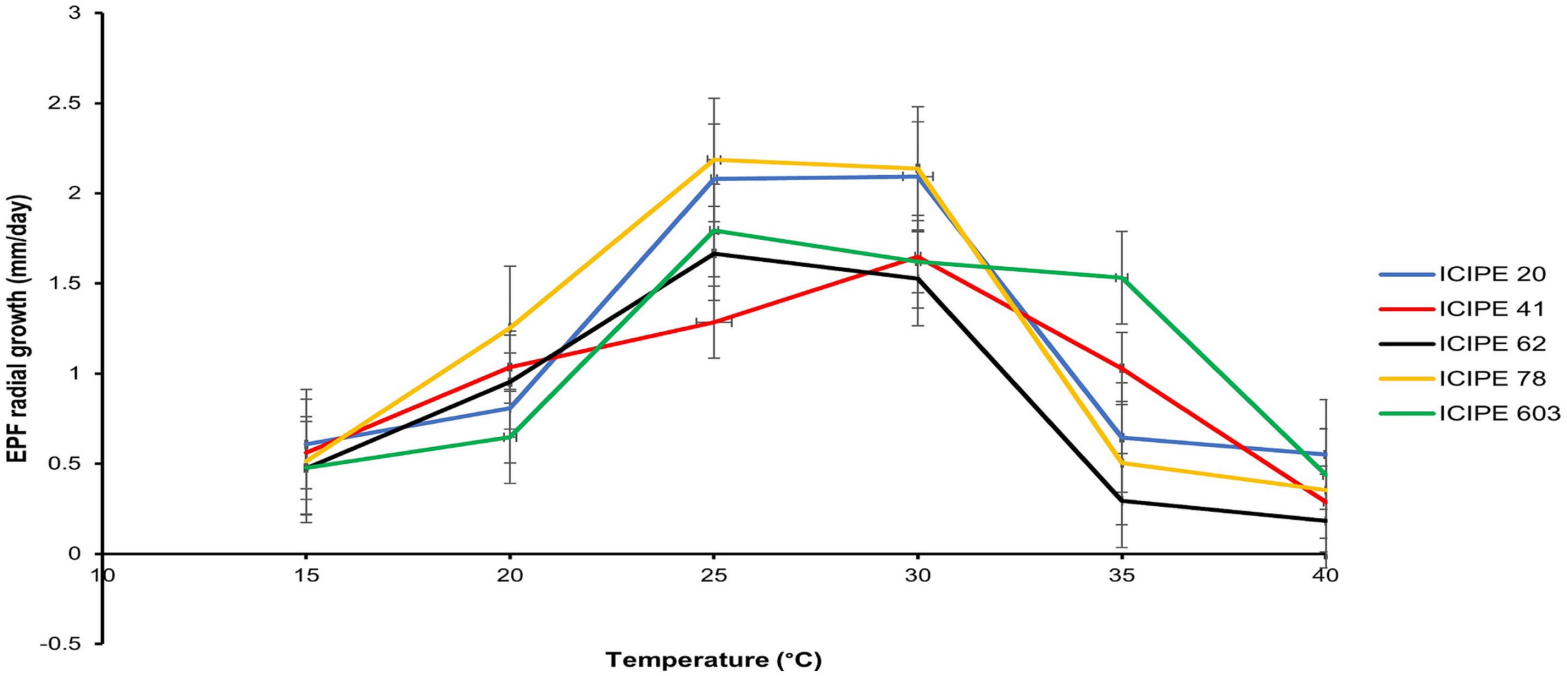
Comparison of the relative growth rates of the SIT-compatible *Metarhizium anisopliae* strains (ICIPE 20, ICIPE 41, ICIPE 62 and ICIPE 78) and *Beauveria bassiana* ICIPE 603, at temperatures ranging from 15 to 40°C (*n* = 120).

The interaction of the radial growth of the SIT-compatible EPF, *M. anisopliae* strains (ICIPE 20, ICIPE 41, ICIPE 62, ICIPE 78) and *B. bassiana* ICIPE 603 at the different temperature regimes (15–40°C) indicated significant affect the temperature on the radial growth parameters of these strains (df = 20, *F* = 3.93, *p* = <0.001) (Figure 2). Among the strains, *M. anisopliae* ICIPE 78 exhibited the fastest conidia growth at 25°C measuring 2.19 ± 0.16 mm/day, while the slowest growth (0.30 ± 0.0 mm/day) was observed in *M. anisopliae* ICIPE 62 at 40°C.

The germination rates across the set temperatures for the selected isolates differed significantly (*χ*^2^ = 165.42, df = 20, *p* < 0.0001) (Table 2). In addition, the spore yield of the SIT-compatible EPF strains varied significantly across the various temperatures (*χ*^2^ = 112.82, df = 20, *p* < 0.0001), with the lowest yield observed at 40°C while the highest yield was obtained at 25°C (Table 3).

**Table 2:** SIT-compatible entomopathogenic fungi strains germination rates across the various temperature regimes.

Germination rate (%)						
Isolate	15°C	20°C	25°C	30°C	35°C	40°C
ICIPE 20	37.71 ± 2.69 ab	78.33 ± 1.83 def	90.38 ± 1.84 ef	99.63 ± 0.12 f	80.71 ± 2.06 def	32.86 ± 1.33 a
ICIPE 41	47.96 ± 1.68 abc	79.38 ± 2.80 def	94.58 ± 2.45 f	98.17 ± 0.75 f	63.67 ± 0.89 cde	39.13 ± 1.94 ab
ICIPE 62	85 ± 1.24 ef	95.33 ± 2.04 f	84.5 ± 3.51 ef	76.63 ± 0.82 def	75.58 ± 2.81 def	32.96 ± 4.85 a
ICIPE 78	32.83 ± 1.40 a	56.86 ± 3.09 bcd	90.96 ± 1.94 f	94.30 ± 1.07 f	57.13 ± 4.13 bcd	46.5 ± 4.20 abc
ICIPE 603	78.71 ± 3.65 def	79.71 ± 2.28 def	95.08 ± 2.01 f	96.08 ± 1.66 f	84.21 ± 0.79 ef	34.21 ± 2.57 a

The values represent mean ± SE. Means within a column followed by the same letters are not significantly different (ANOVA followed by Tukey’s HSD post hoc test, *p* < 0.05, *n* = 120).

**Table 3:** SIT-compatible entomopathogenic fungi strains spore yield harvested after 14 days, across the various temperature regimes.

Spore yield (× 107 conidia/mL)						
Isolate	15°C	20°C	25°C	30°C	35°C	40°C
ICIPE 20	8.13 ± 1.68 abc	11 ± 5.13 abcde	38.63 ± 1.50 hij	26.88 ± 3.62 efghi	17.25 ± 0.18 bcdefg	1.88 ± 0.09 a
ICIPE 41	6.63 ± 1.68 ij	18.38 ± 0.9 kl	23 ± 2.65 kl	10.75 ± 4.07 l	3.13 ± 1.15 jk	2.38 ± 0.44 ghi
ICIPE 62	31.88 ± 0.8 fghi	22.13 ± 1.15 cdefgh	46.25 ± 3.18 ij	25.5 ± 3.36 defghi	6.88 ± 0.80 ab	3.93 ± 0.23 ab
ICIPE 78	4.38 ± 1.86 ab	4.75 ± 0.71 ab	14.5 ± 0.35 abcdef	14.5 ± 6.19 abcdef	3.38 ± 0.09 a	2.63 ± 0.09 a
ICIPE 603	5 ± 0.88 ab	5.63 ± 0.09 ab	7.25 ± 0.71 abc	9.88 ± 0.09 abcd	4.63 ± 0.09 ab	1.43 ± 0.05 a

The values represent mean ± SE. Means within a column followed by the same letters are not significantly different (ANOVA followed by Tukey’s HSD post hoc test, *p* < 0.05, *n* = 120).

**Figure 3:**
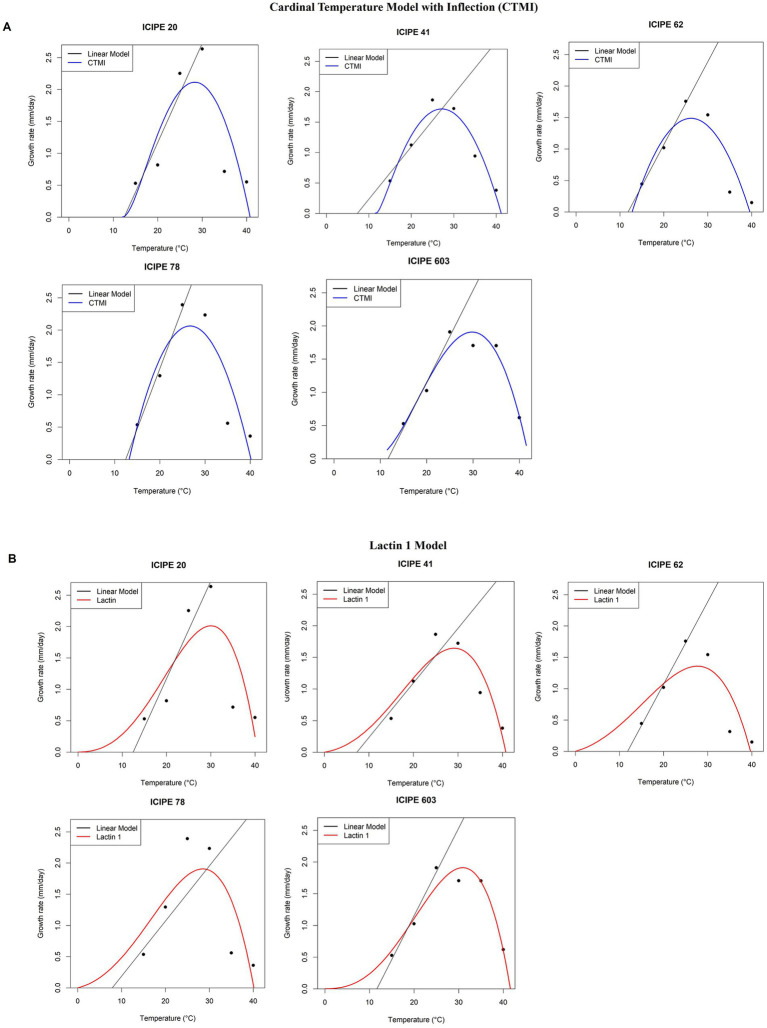
Relationship between radial growth rates of the SIT-compatible EPF and temperature. **(A)** Relative radial growth rates of *M. anisopliae* ICIPE 20, 41, 62, 78 and *B. bassiana* 603 using the Cardinal Temperature Model with Inflection (CTMI) between 15 and 40°C. **(B)** Relative radial growth rates of *M. anisopliae* ICIPE 20, 41, 62, 78 and *B. bassiana* 603 using the Lactin 1 between 15–40°C. The CTMI models are represented by the blue continuous line, Lactin 1 models are represented by the red continuous line while the linear model is represented by the black continuous line (*n* = 120; Tukey’s HSD test).

The fitted models for the absolute radial growth of the SIT-compatible EPF are depicted in Figures 3A,B. The cardinal temperature model with inflection (CTMI) model (Figure 3A) predicted lower temperature thresholds (*Tmin*) of 9.0°C, −3.7°C, 8.1°C, 13.1°C and 9.8°C for *M. anisopliae* ICIPE 20, ICIPE 41, ICIPE 62, ICIPE 78 and *B. bassiana* ICIPE 603, respectively (Table 4). The upper thresholds (*Tmax*) were 35.8°C, 35.2°C, 35.6°C, 40.2°C and 45.4°C for *M. anisopliae* ICIPE 20, ICIPE 41, ICIPE 62, ICIPE 78 and *B. bassiana* ICIPE 603, respectively (Table 4). Furthermore, the optimum thresholds (*Topt*) were 29.5°C, 30.8°C, 27.5°C, 26.7°C and 31.1°C for *M. anisopliae* ICIPE 20, ICIPE 41, ICIPE 62, ICIPE 78 and *B. bassiana* ICIPE 603, respectively (Table 4). Moreover, a positive relationship between temperature and absolute radial growth rate was observed (*R*^2^ = 0.87, *R*^2^ = 0.99, *R*^2^ = 0.99, *R*^2^ = 0.77 and *R*^2^ = 0.84 for *M. anisopliae* ICIPE 20, ICIPE 41, ICIPE 62, ICIPE 78 and *B. bassiana* ICIPE 603, respectively) (Table 4).

**Table 4:** Cardinal estimates from the different models for conidial germination of the SIT-compatible EPF strains.

Model^a^	Parameter^b^	ICIPE 20	ICIPE 41	ICIPE 62	ICIPE 78	ICIPE 603
Lactin 1	*a*	0.000060	0.000033	0.000023	0.0000037	0.0000054
	$T_{\text{min}}$ (°C)	−2.4	−9.35	−1.49	−1.07	−1.73
	$T_{\text{max}}$ (°C)	40.57	40.75	39.7	40.13	41.59
	*R*^2^ *(ln)*	0.88	0.75	0.99	0.98	0.95
	*R*^2^ (Lactin 1)	0.87	0.93	12.63	16.49	0.81
	AIC *(ln)*	6.17	5.11	−5.03	−0.70	0.12
	AIC (Lactin 1)	17.81	7.44	−6.05	15.71	2.56
CTMI	$\mu_{\mathrm{opt}}$ (mm day^−1^)	2.11	1.72	1.49	2.06	1.91
	$T_{\text{min}}$ (°C)	12.10	11.63	12.77	13.19	8.29
	$T_{\mathrm{opt}}$ (°C)	28.33	27.16	26.18	26.68	29.81
	$T_{\text{max}}$ (°C)	40.78	41.18	39.60	40.17	42.12
	*R*^2^ *(ln)*	0.74	0.75	0.99	0.78	0.71
	*R*^2^ (CTMI)	04	0.99	0.99	0.77	0.81
	AIC *(ln)*	6.68	5.11	−5.03	7.44	5.96
	AIC (CTMI)	18.27	4.24	11.76	15.63	3.21

a Temperature–dependent model: Lactin 1, and CMTI (cardinal temperature model with inflection).

b Model’s parameters: *b* are curve–fitting parameters; $T_{\text{min}}$ , $T_{\mathrm{opt}}$ _,_ and $T_{\text{max}}$ are minimum, optimum, and maximum temperatures (°C); $\mu_{\mathrm{opt}}$ is maximal conidial germination (mm day^−1^) at $T_{\mathrm{opt}}$ . The models are reparametrized to provide $T_{\mathrm{opt}}$ . *AIC*, Akaike information criterion; *R*^2^ = adjusted R–squared; *ln* = Linear model.

On the other hand, the Lactin 1 model (Figure 3B) predicted lower temperature threshold (*Tmin*) of 9.6°C, 4.0°C, 5.3°C, −1.1°C and − 1.8°C for *M. anisopliae* ICIPE 20, ICIPE 41, ICIPE 62, ICIPE 78 and *B. bassiana* ICIPE 603, respectively (Table 4). The upper thresholds (*Tmax*) were 36.0°C, 35.8°C, 35.6°C, 40.0°C and 43.7°C for *M. anisopliae* ICIPE 20, ICIPE 41, ICIPE 62, ICIPE 78 and *B. bassiana* ICIPE 603, respectively (Table 4).

### 3.3 Horizontal transmission, spore retention of the selected SIT-compatible EPF isolates by *Glossina pallidipes* and its effects on *Glossina pallidipes* survival

**Figure 4:**
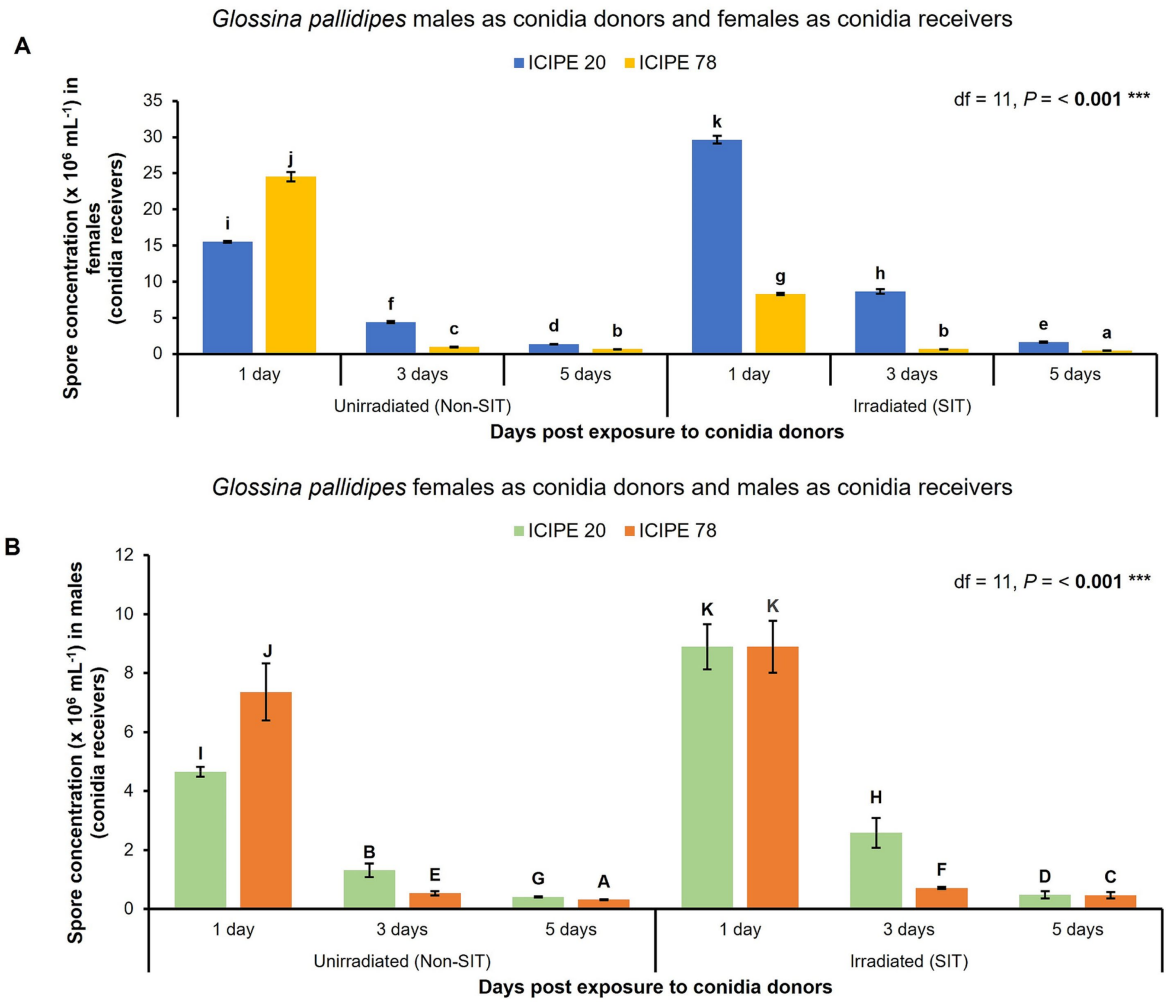
Horizontal transmission of SIT-compatible *Metarhizium anisopliae* conidia in *Glossina pallidipes* (analysis by ANOVA followed by Tukey’s HSD *post hoc* test; *p* > 0.05, *** *p* < 0.001, *n* = 10, replicated thrice). Error bars indicate the standard error of the mean and the bars with different lowercase letters are significantly different from each other. **(A)** Variation of *M. anisopliae* (ICIPE 20 and ICIPE 78) conidia transmission from irradiated and unirradiated *G. pallidipes* males (conidia donors) to unirradiated virgin *G. pallidipes* females (conidia receivers) across time. **(B)** Variation of *M. anisopliae* (ICIPE 20 and ICIPE 78) conidia transmission from EPF-exposed *G. pallidipes* females (conidia donors) to irradiated and unirradiated *G. pallidipes* males (conidia receivers) across time.

Only *M. anisopliae* ICIPE 20 and ICIPE 78, which emerged as the most thermal stable EPF for incorporation with SIT were used in the horizontal transmission, spore retention and *G. pallidipes* survival bioassays. Consequently, when the irradiated-EPF-exposed *G. pallidipes* males were used as conidia donors, there was significant difference in spore acquisition and retention among the conidia receivers (*G. pallidipes* females) (df = 11, *p* < 0.001) (Figure 4A). Additionally, 5 days post-exposure to the irradiated EPF-challenged males (conidia donors), the females (conidia receivers) still retained a high quantity of spores (> 1.6 × 10^6^ conidia/mL) for horizontal transfer among their conspecific species. Similarly, when the *G. pallidipes* females were used as conidia donors and the irradiated *G. pallidipes* males as conidia receivers, there was significant difference in the conidia uptake and retention among the conidia receivers (irradiated *G. pallidipes* males) (df = 11, *p* < 0.001) (Figure 4B). Moreover, 5 days post exposure to the EPF-exposed females, the irradiated males still had sufficient spores (> 0.48 × 10^6^ conidia/mL) to transmit horizontally to their mates. Both the *G. pallidipes* males (conidia donors) and females (conidia receivers) were susceptible to the selected SIT-compatible EPF isolate infection and there was no significant difference (*F* = 1.79, Dfn = 3, Dfd = 3, *p* = 0.6438) attributed to the sex of the *G. pallidipes*.

### 3.4 Effect of the SIT-compatible entomopathogenic fungal isolates on *Glossina pallidipes* survival rates

**Figure 5:**
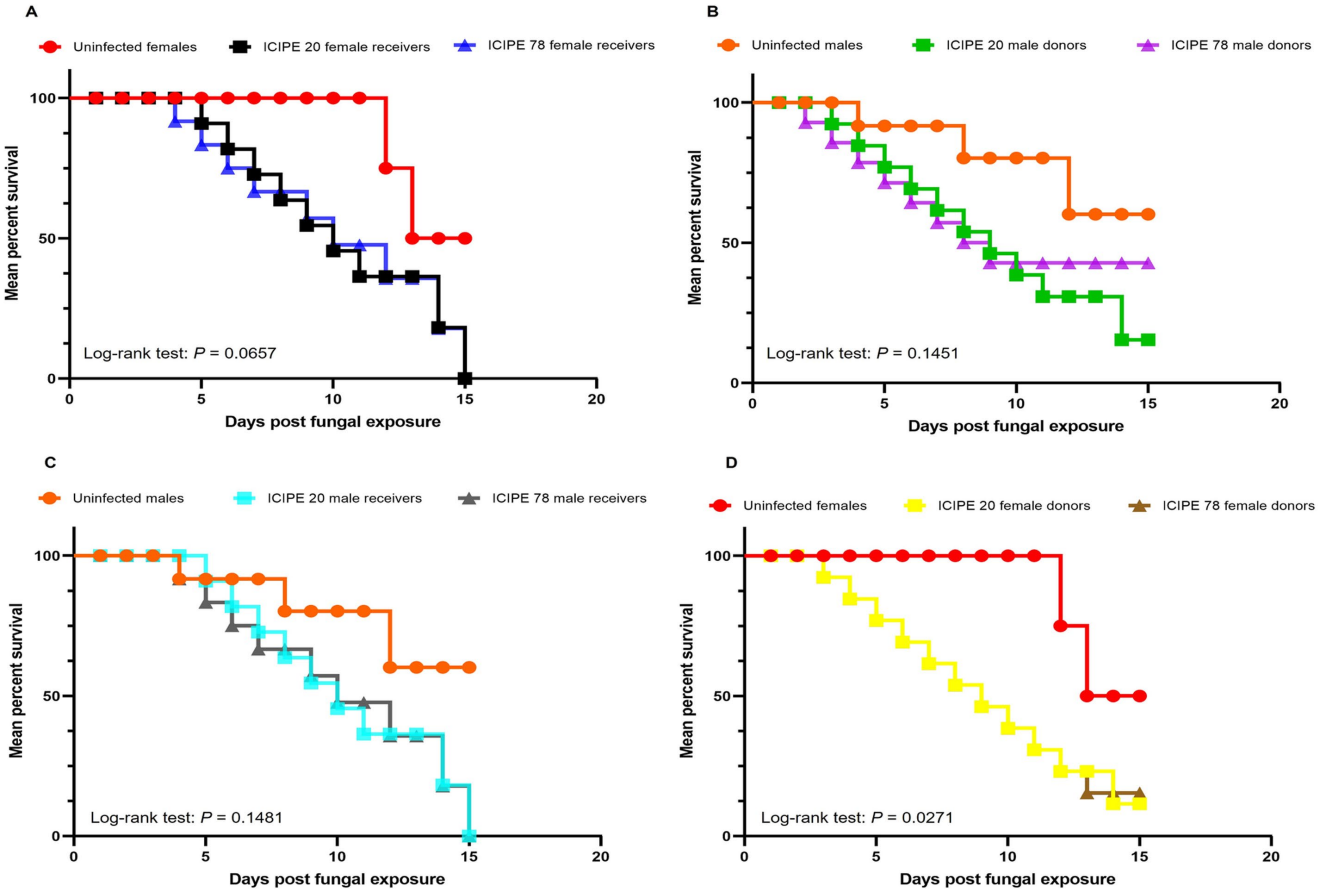
Kaplan–Meier curve illustrating survivorship over time. **(A)** Survival curves of the conidia donors (virgin *G. pallidipes* females) over time. **(B)** Survival curves of the conidia donors (irradiated *G. pallidipes* males) over time. **(C)** Survival curves of the conidia receivers (irradiated *G. pallidipes* males) over time. **(D)** Survival curves of the conidia receivers (virgin *G. pallidipes* females) over time after exposure to irradiated-EPF-exposed *G. pallidipes* males (Mantel-Cox log-rank *χ*^2^ test, *p* < 0.05, *n* = 30).

When the irradiated-EPF-exposed *G. pallidipes* males were used as conidia donors and the virgin *G. pallidipes* females were the conidia receivers, there was no significant difference in the survival rates of conidia receivers (unirradiated *G. pallidipes* females that had received conidia from EPF exposed irradiated males) (Log-rank (Mantel-Cox) test, *χ*^2^ = 5.447, df = 2, *p* = 0.0657) (Figure 5A). Similarly, there was no significant difference in the survival rates of the irradiated-EPF-exposed *G. pallidipes* males (conidia donors) (Log-rank (Mantel-Cox) test, *χ*^2^ = 3.86, df = 2, *p* = 0.1451) (Figure 5B).

On the other hand, when EPF-exposed *G. pallidipes* females were used as the conidia donors and the irradiated EPF-free *G. pallidipes* males as conidia receivers, there was no significant difference in the survival rates of the conidia receivers (irradiated *G. pallidipes* males that had received conidia from EPF exposed virgin females) (Log-rank (Mantel-Cox) test, *χ*^2^ = 3.82, df = 2, *p* = 0.1481) (Figure 5C). However, there was significant difference in the survival rates of the EPF-exposed *G. pallidipes* females (conidia donors) (Log-rank (Mantel-Cox) test, *χ*^2^ = 7.22, df = 2, *p* = 0.0271) (Figure 5D). Overall, there was no statistically significant difference in survival rates between the conidia donors and receivers as influenced by the SIT-compatible EPF strains (Log-rank (Mantel-Cox) test, *χ*^2^ = 11.96, df = 9, *p* = 0.2153).

**Figure 6:**
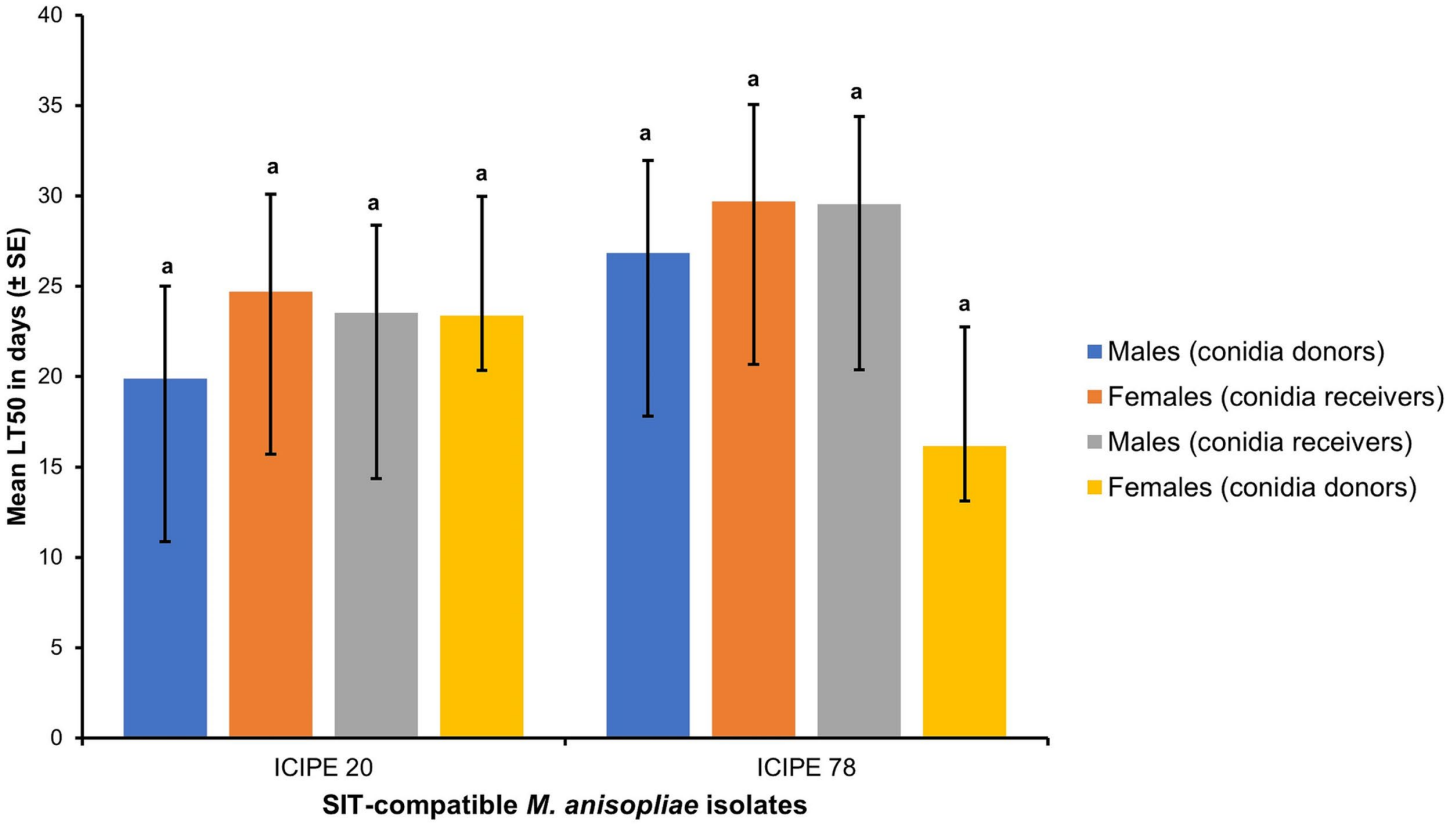
Mean lethal time (LT_50_) between *Glossina pallidipes* conidia donors and conidia receivers (both male and female) (*p* > 0.05, *n* = 10). Error bars indicate the standard error of the mean and the bars with different lowercase letters are significantly different from each other.

There was no sex dimorphism observed in males and females of *G. pallidipes* after the acquisition of conidia (Log-rank (Mantel-Cox) test, *χ*^2^ = 11.96, df = 9, *p* = 0.2153). Furthermore, there were no significant difference in the longevity of the *G. pallidipes* conidia donors and conidia receivers (both male and female) (*t* = 0.42, df = 6, *p* = 0.6888) (Figure 6).

## 4 Discussion

Current vector management options for tsetse flies have had limited success, and chemotherapy-based solutions against trypanosomiasis have led to resistance in targeted trypanosomes (Geerts et al., 2001). Given the substantial economic burden these flies impose on the African continent (Nthiwa et al., 2015), there is an urgent need for more effective solutions. Combining entomopathogenic fungi (EPF) with the sterile insect technique (SIT) within an area-wide Integrated Pest Management (IPM) strategy offers a promising path toward the near eradication of these harmful vectors. This innovative approach could significantly reduce the impact of tsetse flies, leading to improved economic and public health outcomes in affected regions.

In this study, out of the 11 entomopathogenic fungal isolates screened, five isolates (*M. anisopliae* strains ICIPE 20, ICIPE 41, ICIPE 62, ICIPE 78, and *B. bassiana* strain ICIPE 603) were identified as SIT-compatible/tolerant. This selection was based on their LT_50_ values *vis-à-vis* their killing rates, tolerance to temperature, radial growth, and conidia production. Among these strains, *M. anisopliae* ICIPE 20 and ICIPE 78 demonstrated the highest tolerance to temperature, exhibited the fastest radial growth, and had the greatest conidia production. From the temperature-dependent modelling, the optimal temperature was 26.7–31.1°C and it is at this range that *M. anisopliae* ICIPE 20 and ICIPE 78 were at their peak in terms of absolute radial growth. Pagabeleguem et al. (2016) studied the survival and fecundity of *Glossina palpalis gambiensis* strains under various conditions and observed that no tsetse species survived beyond 32°C. Moreover, with the preferred temperature range for tsetse survival being 16–32°C (Are and Hargrove, 2020), it could be advantageous to integrate these two potent isolates in the vector management since this is within the optimal temperature (*Topt*) range for radial growth of *M. anisopliae* ICIPE 20 and ICIPE 78. The observed thermal range is in congruence with observations by Hywel-Jones and Gillespie (1990), who reported that the highest germination rate among *M. anisopliae* strains is attained between 25 and 30°C. Even though we observed delayed conidial germination at 15°C by *M. anisopliae* ICIPE 20 and ICIPE 78, and low germination collectively observed at 35 and 40°C, ≥ 90% of the conidia germinated at 20, 25 and 30°C. This intraspecific variation in germination among SIT-compatible EPF strains could conclusively be attributed to the prevailing temperatures (Agbessenou et al., 2021; De Croos and Bidochka, 1999). Overall, the maximum conidial germination at the most optimal temperature was observed in *M. anisopliae* ICIPE 20 and ICIPE 78, respectively.

Consequently, our findings indicate that *M. anisopliae* ICIPE 20 and ICIPE 78 could be deployed for the management of tsetse flies populations, in their natural habitats. We have demonstrated that *M. anisopliae* ICIPE 20 and ICIPE 78 are able to tolerate the prevailing environmental temperatures, thereby maintaining their viability and efficacy rates against *G. pallidipes* thus making them both potent and SIT-compatible. As such, *M. anisopliae* ICIPE 20 and ICIPE 78 were deemed the most compatible for incorporation into *G. pallidipes*-IPM either individually or in combination with tsetse SIT, for the sustainable management of *G. pallidipes*.

Our key hypothesis was to validate EPF strains that presented moderate lethal times to give sufficient time for the sterilised and infected males to mate with the wild virgin females while transmitting the conidia to the conspecific mates before they both eventually succumb to EPF infection. Our findings demonstrated that *M. anisopliae* ICIPE 20 and ICIPE 78 not only have moderate LT_50_ times, but their inoculum can be acquired and transmitted horizontally over a short period either during attempting for mating, mating, or fighting. The ability of the females to mate once in their lifetime in most of tsetse species when living on or near the males (Leak, 1998) presents a viable opportunity to reduce the population through SIT and EPF horizontal conidia transfer. This study focused on using EPF since they present a much greater opportunity as biological control agents of tsetse due to their mode of action of infection through the cuticle as compared to other pathogens (viruses, nematodes, bacteria, protozoans) that must be ingested first (Maniania, 1998). Moreover, our results have demonstrated that both male and female *G. pallidipes* can transmit the EPF conidia and there was no disparity between the males and females in terms of survival after exposure to EPF. The lifespan of a female tsetse is around 4 months (under feeding conditions), and she mates just once when living on or near a target species. When a male tsetse perceives a pheromone on the female’s body, the males initiate pairing, and the copulation can last up to 2 hours (Wall and Langley, 1993). Therefore, through horizontal transmission of SIT-compatible fungal isolates (*M. anisopliae* ICIPE 20 and ICIPE 78), the population of the flies could be effectively and sustainably suppressed while reducing the misuse of synthetic pesticides in these important vectors management.

During EPF infection, insects will ordinarily undergo some physiological and behavioural changes such as fever, loss of appetite, reduction in blood meal intake, and reduction in vector competence as reported in desert locusts (Hunt and Charnley, 2011), *G. morsitans morsitans* (Maniania et al., 2013), *G. f. fuscipes* (Wamiti et al., 2018) and in mosquitoes (Fang et al., 2011). We observed that *G. pallidipes* infected with the SIT-compatible *M. anisopliae* ICIPE 20 and ICIPE 78 were still able to feed 5 days post fungus exposure, although their activities and feeding behaviour were reduced. This result was similar to the findings reported by Wamiti et al. (2018) on *G. f. fuscipes* flies that continued to feed for 3 days following the fungus infection; and Baleba et al. (2021) on stable fly, *Stomoxys calcitrans* (Diptera: Muscidae). This was an important finding as it gives credence to the use of EPF with longer LT_50_ activity to complement SIT. In this regard, the use of SIT-compatible fungal isolates *M. anisopliae* ICIPE 20 and ICIPE 78 may ensure that; (i) the infected males would be active for a longer period giving them sufficient time to transfer conidia to other tsetse either during grooming, mating, or fights; (ii) the physiology of the infected males might not be affected very fast hence maintaining the required competitiveness as with the non-lab-reared males and; (iii) the females that will get infected with conidia through horizontal transfer would continue spreading the conidia to other males (both SIT released and wild) for a longer period (so long as there is contact). In the end, the EPF infection would lead to the gradual collapse of the entire population as females, having mated with males whose seminal fluid is sterile, would produce no viable offspring, compounded by the induced mortality from the SIT-compatible fungi.

## 5 Conclusion

This study has demonstrated the compatibility of *M. anisopliae* ICIPE 20 and ICIPE 78 with SIT for the management of *G. pallidipes*. The selected strains had moderate lethal times (LT_50_) and had optimal radial growth rates (germination, mycelial growth and sporulation) within the tsetse survival temperature ranges. Additionally, our findings have demonstrated that both *M. anisopliae* ICIPE 20 and ICIPE 78 conidia can be acquired and horizontally transmitted by both *G. pallidipes* males and females, and conidia retention among the infected conidia receivers could still be high 5 days post-exposure to an EPF-infected mate. Hence, *M. anisopliae* ICIPE 20 and ICIPE 78 could be developed as biopesticides products and rolled out in an area-wide IPM program against *G. pallidipes* in synergy with sterile insect technique. However, to effectively utilise these two biopesticides, field validation trials across the different tsetse endemic areas need to be conducted. Moreover, abiotic factors such as humidity, solar radiation (UV light), rainfall, soil pH, soil type and texture, and wind can significantly influence the validation of our biopesticide products (*M. anisopliae* ICIPE 20 and ICIPE 78). To enhance the effectiveness of *M. anisopliae* ICIPE 20 and ICIPE 78 biopesticides in the field, it would be essential to implement strategies that mitigate the adverse effects of these abiotic factors. Through follow-up experiments and by optimising environmental conditions through careful planning and adaptive management, we can greatly improve the performance and reliability of these biopesticides. Additionally, the infected host (*G. pallidipes*) is not a passive participant in the EPF infection process. As such, *G. pallidipes* might initiate cellular and humoral responses against the EPF in a bid to resist the EPF that needs to be investigated. The EPF may also elicit gut/insect microbiota in response to the fungal infection; in this regard, 16S metagenomics needs to be conducted on both pre-and post-fungal infected tsetse to understand these mechanisms and the role of the gut microbiota in the EPF infective process. Overall, this novel dual-pronged management approach has a potential of effective management of *G. pallidipes* after field validation.

## Data Availability

The original contributions presented in the study are included in the article/Supplementary material, further inquiries can be directed to the corresponding author.

## References

[ref1] Abbott W. S. (1925). A method of computing the effectiveness of an insecticide. J. Econ. Entomol. 18, 265–267. doi: 10.1093/jee/18.2.265a

[ref2] Agbessenou A. Akutse K. S. Yusuf A. A. Wekesa S. W. Khamis F. M. (2021). Temperature-dependent modelling and spatial prediction reveal suitable geographical areas for deployment of two Metarhizium anisopliae isolates for Tuta absoluta management. Sci. Rep. 11:23346. doi: 10.1038/s41598-021-02718-w, PMID: 34857835 PMC8639720

[ref3] Aksoy S. Buscher P. Lehane M. Solano P. Van Den Abbeele J. (2017). Human African trypanosomiasis control: achievements and challenges. PLoS Negl. Trop. Dis. 11:e0005454. doi: 10.1371/journal.pntd.0005454, PMID: 28426685 PMC5398477

[ref4] Akutse K. S. Subramanian S. Maniania N. Dubois T. Ekesi S. (2020). Biopesticide research and product development in Africa for sustainable agriculture and food security–experiences from the International Centre of Insect Physiology and Ecology (icipe). Front. Sustain. Food Syst. 4:152. doi: 10.3389/fsufs.2020.563016

[ref5] Are E. B. Hargrove J. W. (2020). Extinction probabilities as a function of temperature for populations of tsetse (Glossina spp.). PLoS Negl. Trop. Dis. 14:e0007769. doi: 10.1371/journal.pntd.0007769, PMID: 32379749 PMC7237048

[ref6] Arthurs S. Thomas M. B. (2001). Effects of temperature and relative humidity on sporulation of Metarhizium anisopliae var. acridum in mycosed cadavers of Schistocerca gregaria. J. Invertebr. Pathol. 78, 59–65. doi: 10.1006/jipa.2001.5050, PMID: 11812107

[ref7] Asfaw N. Hiruy B. Worku N. Massebo F. (2022). Evaluating the efficacy of various traps in catching tsetse flies at Nech Sar and maze National Parks, southwestern Ethiopia: an implication for Trypanosoma vector control. PLoS Negl. Trop. Dis. 16:e0010999. doi: 10.1371/journal.pntd.0010999, PMID: 36548437 PMC9822101

[ref8] Baleba S. B. Agbessenou A. Getahun M. N. Akutse K. S. Subramanian S. Masiga D. (2021). Infection of the stable fly, Stomoxys calcitrans, L. 1758 (Diptera: Muscidae) by the entomopathogenic fungi Metarhizium anisopliae (Hypocreales: Clavicipitaceae) negatively affects tts survival, feeding propensity, fecundity, fertility, and fitness parameters. Front. Fungal Biol. 2:637817. doi: 10.3389/ffunb.2021.637817 37744116 PMC10512350

[ref9] Bardosh K. Waiswa C. Welburn S. C. (2013). Conflict of interest: use of pyrethroids and amidines against tsetse and ticks in zoonotic sleeping sickness endemic areas of Uganda. Parasit. Vectors 6, 1–15. doi: 10.1186/1756-3305-6-204 23841963 PMC3711891

[ref10] Beadell J. S. Hyseni C. Abila P. P. Azabo R. Enyaru J. C. Ouma J. O.. (2010). Phylogeography and population structure of Glossina fuscipes fuscipes in Uganda: implications for control of tsetse. PLoS Negl. Trop. Dis. 4:e636. doi: 10.1371/journal.pntd.0000636, PMID: 20300518 PMC2838784

[ref11] Bitew M. Amide Y. Zenebe T. Degefu H. (2011). Trypanosome infection rate in Glossina pallidipes and Glossina fuscipes fuscipes in Gojeb Valley, Southwest Ethiopia. Glob. Vet. 6, 131–135.

[ref12] Bugeme D. M. Knapp M. Boga H. I. Wanjoya A. K. Maniania N. K. (2009). Influence of temperature on virulence of fungal isolates of Metarhizium anisopliae and Beauveria bassiana to the two-spotted spider mite Tetranychus urticae. Mycopathologia 167, 221–227. doi: 10.1007/s11046-008-9164-6, PMID: 18987988

[ref13] Büscher P. Cecchi G. Jamonneau V. Priotto G. (2017). Human African trypanosomiasis. Lancet 390, 2397–2409. doi: 10.1016/S0140-6736(17)31510-6 28673422

[ref14] Cecchi G. Mattioli R. C. Slingenbergh J. De La Rocque S. (2008). Land cover and tsetse fly distributions in sub-Saharan Africa. Med. Vet. Entomol. 22, 364–373. doi: 10.1111/j.1365-2915.2008.00747.x, PMID: 18785934

[ref15] Cecchi G. Paone M. Argilés Herrero R. Vreysen M. J. Mattioli R. C. (2015). Developing a continental atlas of the distribution and trypanosomal infection of tsetse flies (Glossina species). Parasit. Vectors 8, 1–10. doi: 10.1186/s13071-015-0898-y 25994757 PMC4448735

[ref16] Ciosi M. Masiga D. K. Turner C. M. (2014). Laboratory colonisation and genetic bottlenecks in the tsetse fly Glossina pallidipes. PLoS Negl. Trop. Dis. 8:e2697. doi: 10.1371/journal.pntd.0002697, PMID: 24551260 PMC3923722

[ref17] De Croos J. N. A. Bidochka M. J. (1999). Effects of low temperature on growth parameters in the entomopathogenic fungus Metarhizium anisopliae. Can. J. Microbiol. 45, 1055–1061. doi: 10.1139/w99-098

[ref18] de Faria M. R. Wraight S. P. (2007). Mycoinsecticides and mycoacaricides: a comprehensive list with worldwide coverage and international classification of formulation types. Biol. Control 43, 237–256. doi: 10.1016/j.biocontrol.2007.08.001

[ref19] Dimbi S. Maniania N. K. Lux S. A. Ekesi S. Mueke J. K. (2003). Pathogenicity of Metarhizium anisopliae (Metsch.) Sorokin and Beauveria bassiana (Balsamo) Vuillemin, to three adult fruit fly species: Ceratitis capitata (Weidemann), C. rosa var. fasciventris Karsch and C. cosyra (Walker) (Diptera: Tephritidae). Mycopathologia 156, 375–382. doi: 10.1023/B:MYCO.0000003579.48647.16, PMID: 14682465

[ref20] Dyck V. A. Hendrichs J. Robinson A. S. (2021). Sterile insect technique: Principles and practice in area-wide integrated pest management. Boca Raton, Florida, USA: Taylor & Francis, 75–112.

[ref004] Elzhov T. V. Mullen K. M. Spiess A. N. Bolker B. (2016). Minpack.lm: R interface to the levenberg-marquardt nonlinear least–squares algorithm. R package version 1. 2–1. https://CRAN.R-project.org/package=minpack.lm

[ref21] Fang W. Vega-Rodríguez J. Ghosh A. K. Jacobs-Lorena M. Kang A. Leger R. J. S. (2011). Development of transgenic fungi that kill human malaria parasites in mosquitoes. Science 331, 1074–1077. doi: 10.1126/science.1199115, PMID: 21350178 PMC4153607

[ref22] Fargues J. Maniania N. K. Delmas J. C. Smits N. (1992). Influence de la température sur la croissance in vitro d'hyphomycètes entomopathogènes. Agronomie 12, 557–564. doi: 10.1051/agro:19920708

[ref23] Franco J. R. Simarro P. P. Diarra A. Jannin J. G. (2014). Epidemiology of human African trypanosomiasis. Clin. Epidemiol. 6, 257–275. doi: 10.2147/CLEP.S39728 25125985 PMC4130665

[ref24] Geerts S. Holmes P. H. Eisler M. C. Diall O. (2001). African bovine trypanosomiasis: the problem of drug resistance. Trends Parasitol. 17, 25–28. doi: 10.1016/S1471-4922(00)01827-4, PMID: 11137737

[ref25] Gindin G. Levski S. Glazer I. Soroker V. (2006). Evaluation of the entomopathogenic fungi Metarhizium anisopliae and Beauveria bassiana against the red palm weevil Rhynchophorus ferrugineus. Phytoparasitica 34, 370–379. doi: 10.1007/BF02981024

[ref26] Goettel M. S. Inglis G. D. (1997). “Fungi: hyphomycetes” in Manual of techniques in insect pathology. ed. Lawrence A. Lacey (Academic press), 213–249.

[ref27] Gooding R. H. Krafsur E. S. (2005). Tsetse genetics: contributions to biology, systematics, and control of tsetse flies. Annu. Rev. Entomol. 50, 101–123. doi: 10.1146/annurev.ento.50.071803.130443, PMID: 15355235 PMC1462949

[ref28] Hajek A. E. St Leger R. J. (1994). Interactions between fungal pathogens and insect hosts. Annu. Rev. Entomol. 39, 293–322. doi: 10.1146/annurev.en.39.010194.001453

[ref29] Hendrichs J. Pereira R. Vreysen M. J. (2021). Area-wide integrated pest management: Development and field application: Taylor & Francis, 1028.

[ref30] Hunt V. L. Charnley A. K. (2011). The inhibitory effect of the fungal toxin, destruxin a, on behavioural fever in the desert locust. J. Insect Physiol. 57, 1341–1346. doi: 10.1016/j.jinsphys.2011.06.008, PMID: 21729702

[ref001] Hussain A. Tian M. Y. He Y. R. Ahmed S. (2009). Entomopathogenic fungi disturbed the larval growth and feeding performance of Ocinara varians (Lepidoptera: Bombycidae) larvae. Insect Science, 16, 511–517. doi: 10.1111/j.1744-7917.2009.01272

[ref31] Hywel-Jones N. L. Gillespie A. T. (1990). Effect of temperature on spore germination in Metarhizium anisopliae and Beauveria bassiana. Mycol. Res. 94, 389–392. doi: 10.1016/S0953-7562(09)80363-8

[ref32] Inglis G. D. Goettel M. S. Butt T. M. Strasser H. E. (2001). “Use of hyphomycetous fungi for managing insect pests” in Fungi as biocontrol agents: progress, problems and potential (Wallingford UK: CABI publishing), 23–69.

[ref33] International Atomic Energy Agency (IAEA) (2024). Sterile insect technique, pest control with sterilised insects. Available at: https://www.iaea.org/topics/sterile-insect-technique (Accessed January 24, 2024).

[ref34] Jamonneau V. Ilboudo H. Kaboré J. Kaba D. Koffi M. Solano P.. (2012). Untreated human infections by Trypanosoma brucei gambiense are not 100% fatal. PLoS Negl. Trop. Dis. 6:e1691. doi: 10.1371/journal.pntd.0001691, PMID: 22720107 PMC3373650

[ref35] Kabayo J. P. (2002). Aiming to eliminate tsetse from Africa. Trends Parasitol. 18, 473–475. doi: 10.1016/S1471-4922(02)02371-1, PMID: 12473355

[ref36] Klassen W. Vreysen M. J. B. (2021). “Area-wide integrated pest management and the sterile insect technique” in Sterile insect technique. eds. Dyck V. A, Hendrichs J. and Robinson A. S (Boca Raton, Florida, USA: CRC Press), 75–112.

[ref37] Knipling E. F. (1955). Possibilities of insect control or eradication through the use of sexually sterile males. J. Econ. Entomol. 48, 459–462. doi: 10.1093/jee/48.4.459

[ref38] Knipling E. F. (1959). Sterile-male method of population control: successful with some insects, the method may also be effective when applied to other noxious animals. Science 130, 902–904. doi: 10.1126/science.130.3380.902 14410136

[ref39] Kvålseth T. O. (1985). Cautionary note about R^2^. Am. Stat. 39, 279–285.

[ref40] Lacey L. A. Kaya H. K. (Eds.) (2007). Field manual of techniques in invertebrate pathology: Application and evaluation of pathogens for control of insects and other invertebrate pests. Dordrecht, Netherlands: Springer Science & Business Media.

[ref41] Lactin D. J. Holliday N. J. Johnson D. L. Craigen R. (1995). Improved rate model of temperature-dependent development by arthropods. Environ. Entomol. 24, 68–75. doi: 10.1093/ee/24.1.68

[ref42] Langley P. A. (1989). Laboratory colonization of the tsetse fly Glossina pallidipes Austen (Diptera: Glossinidae) using an in vitro feeding method. Bull. Entomol. Res. 79, 429–435. doi: 10.1017/S0007485300018435

[ref43] Langley P. A. Curtis C. F. Brady J. (1974). The viability, fertility and behaviour of tsetse flies (Glossina morsitans) sterilized by irradiation under various conditions. Entomol. Exp. Appl. 17, 97–111. doi: 10.1111/j.1570-7458.1974.tb00323.x

[ref44] Leak S. G. A. (1998). Tsetse biology and ecology: Their role in the epidemiology and control of trypanosomosis. Oxford and New York: CABI Publishing, 568.

[ref45] Mangiafico S. (2020). Rcompanion: functions to support extension education program evaluation. R package version 2.3.25.

[ref002] Maniania N. K. (1992). Pathogenicity of entomogenous fungi (Hyphomycetes) to larvae of the stem borers, Chilo partellus Swinhoe and Busseola fusca Fuller. Int. J. Trop. Insect Sci. 13, 691–696. doi: 10.1017/S1742758400007918

[ref46] Maniania N. K. (1994). A laboratory technique for infecting adult tsetse with a fungal pathogen. Int. J. Trop. Insect Sci. 15, 421–426. doi: 10.1017/S1742758400015769

[ref47] Maniania N. K. (1998). A device for infecting adult tsetse flies, Glossina spp., with an entomopathogenic fungus in the field. Biol. Control 11, 248–254. doi: 10.1006/bcon.1997.0580

[ref48] Maniania N. K. (2002). A low-cost contamination device for infecting adult tsetse flies, Glossina spp., with the entomopathogenic fungus Metarhizium anisopliae in the field. Biocontrol Sci. Tech. 12, 59–66. doi: 10.1080/09583150120110662

[ref49] Maniania N. K. Ekesi S. Odulaja A. Okech M. A. Nadel D. J. (2006). Prospects of a fungus-contamination device for the control of tsetse fly Glossina fuscipes fuscipes. Biocontrol Sci. Tech. 16, 129–139. doi: 10.1080/09583150500258503

[ref50] Maniania N. K. Okech M. A. Adino J. O. Opere J. O. Ekesi S. (2013). Transfer of inoculum of Metarhizium anisopliae between adult Glossina morsitans morsitans and effects of fungal infection on blood feeding and mating behaviors. J. Pest. Sci. 86, 285–292. doi: 10.1007/s10340-012-0473-7, PMID: 23687485 PMC3656219

[ref51] Merkle E. You D. (2020). Nonnest2: tests of non–nested models. R package version 0.5–5.

[ref52] Meyling N. V. Eilenberg J. (2007). Ecology of the entomopathogenic fungi Beauveria bassiana and Metarhizium anisopliae in temperate agroecosystems: potential for conservation biological control. Biol. Control 43, 145–155. doi: 10.1016/j.biocontrol.2007.07.007

[ref53] Miller C. D. Rangel D. Braga G. U. Flint S. Kwon S. I. Messias C. L.. (2004). Enzyme activities associated with oxidative stress in Metarhizium anisopliae during germination, mycelial growth, and conidiation and in response to near-UV irradiation. Can. J. Microbiol. 50, 41–49. doi: 10.1139/w03-097, PMID: 15052320

[ref54] Molnar I. Gibson D. M. Krasnoff S. B. (2010). Secondary metabolites from entomopathogenic Hypocrealean fungi. Nat. Prod. Rep. 27, 1241–1275. doi: 10.1039/c001459c, PMID: 20601982

[ref55] Morrison L. J. MacLeod A. (2011). African trypanosomiasis. Parasite Immunol. 33, 421–422. doi: 10.1111/j.1365-3024.2011.01302.x, PMID: 21609334 PMC3443387

[ref56] Mutika G. N. Parker A. G. (2014). Tolerance of low temperature and sterilizing irradiation in males of Glossina pallidipes (Diptera: Glossinidae). J. Insect Sci. 14:262. doi: 10.1093/jisesa/ieu124 25527576 PMC5634028

[ref57] Nthiwa D. M. Odongo D. O. Ochanda H. Khamadi S. Gichimu B. M. (2015). Trypanosoma infection rates in Glossina species in Mtito Andei division, Makueni County, Kenya. J. Parasitol. Res. 2015:607432. doi: 10.1155/2015/607432 26617992 PMC4649094

[ref58] Nugroho I. bin Ibrahim Y. (2007). Efficacy of laboratory prepared wettable powder formulation of entomopathogenous fungi Beauveria bassiana, Metarhizium anisopliae and Paecilomyces fumosoroseus against the Polyphagotarsonemus latus (Bank)(Acari: Tarsonemidae)(broad mite) on Capsicum annum (chilli). J. Biosci. 18, 1–11.

[ref59] Okello I. Mafie E. Eastwood G. Nzalawahe J. Mboera L. E. (2022). African animal trypanosomiasis: a systematic review on prevalence, risk factors and drug resistance in sub-Saharan Africa. J. Med. Entomol. 59, 1099–1143. doi: 10.1093/jme/tjac018, PMID: 35579072

[ref60] Oluwafemi R. A. Ilemobade A. A. Laseinde E. A. O. (2007). The impact of African animal trypanosomosis and tsetse on the livelihood and wellbeing of cattle and their owners in the BICOT study area of Nigeria. Sci. Res. Essays 2, 380–383.

[ref61] Omuse E. R. Niassy S. Wagacha J. M. Ong’amo G. O. Azrag A. G. Dubois T. (2022). Suitable models to describe the effect of temperature on conidial germination and mycelial growth of Metarhizium anisopliae and Beauveria bassiana. Biocontrol Sci. Tech. 32, 281–298. doi: 10.1080/09583157.2021.1993133

[ref62] Pagabeleguem S. Ravel S. Dicko A. H. Vreysen M. J. Parker A. Takac P.. (2016). Influence of temperature and relative humidity on survival and fecundity of three tsetse strains. Parasit. Vectors 9, 1–13. doi: 10.1186/s13071-016-1805-x 27682638 PMC5041576

[ref63] Pitt J. I. Hocking A. D. (2009). Fungi and food spoilage. 3rd Edn. Cham, Switzerland: Springer.

[ref64] R Core Team (2020). R: a language and environment for statistical computing. R software version 4.0.3: R Foundation for Statistical Computing.

[ref65] Rath A. C. (2000). The use of entomopathogenic fungi for control of termites. Biocontrol Sci. Tech. 10, 563–581. doi: 10.1080/095831500750016370

[ref66] Rosso L. Lobry J. R. Bajard S. Flandrois J. P. (1995). Convenient model to describe the combined effects of temperature and pH on microbial growth. Appl. Environ. Microbiol. 61, 610–616. doi: 10.1128/aem.61.2.610-616.1995, PMID: 16534932 PMC1388350

[ref67] Shapiro S. S. Wilk M. B. (1965). An analysis of variance test for normality (complete samples). Biometrika 52, 591–611. doi: 10.1093/biomet/52.3-4.591

[ref68] Sharififard M. Mossadegh M. S. Vazirianzadeh B. Mahmoudabadi A. Z. (2011). Laboratory evaluation of pathogenicity of entomopathogenic fungi, Beauveria bassiana (Bals.) Vuill. And Metarhizium anisopliae (Metch.) Sorok. To larvae and adults of the house fly, Musca domestica L.(Diptera: Muscidae). Asian J. Biol. Sci. 4, 128–137. doi: 10.3923/ajbs.2011.128.137

[ref69] St Leger R. J. (1995). The role of cuticle-degrading proteases in fungal pathogenesis of insects. Can. J. Bot. 73, 1119–1125. doi: 10.1139/b95-367

[ref70] Therneau T. (2015). A package for survival analysis in S. Version 2.38. Available at: https://CRAN.R-project.org/package=survival.

[ref71] Thomas M. B. Blanford S. Lomer C. J. (1997). Reduction of feeding by the variegated grasshopper, Zonocerus variegatus, following infection by the fungal pathogen, Metarhizium flavoviride. Biocontrol Sci. Tech. 7, 327–334. doi: 10.1080/09583159730730

[ref003] Vidal C. Fargues J. Lacey L. A. (1997). Intraspecific variability of Paecilomyces fumosoroseus: effect of temperature on vegetative growth. Journal of Invertebrate Pathology. 70, 18–26. doi: 10.1006/jipa.1997.4658, PMID: 20601982

[ref72] Vreysen M. J. Saleh K. M. Ali M. Y. Abdulla A. M. Zhu Z. R. Juma K. G.. (2000). Glossina austeni (Diptera: Glossinidae) eradicated on the island of Unguja, Zanzibar, using the sterile insect technique. J. Econ. Entomol. 93, 123–135. doi: 10.1603/0022-0493-93.1.123, PMID: 14658522

[ref73] Vuong Q. (1989). Likelihood ratio tests for model selection and non–nested hypotheses. Econometrica 57, 307–333. doi: 10.2307/1912557

[ref74] Wall R. Langley P. A. (1993). The mating behaviour of tsetse flies (Glossina): a review. Physiol. Entomol. 18, 211–218. doi: 10.1111/j.1365-3032.1993.tb00470.x

[ref75] Wamiti L. G. Khamis F. M. Abd-alla A. M. Ombura F. L. Akutse K. S. Subramanian S.. (2018). Metarhizium anisopliae infection reduces Trypanosoma congolense reproduction in Glossina fuscipes fuscipes and its ability to acquire or transmit the parasite. BMC Microbiol. 18:142. doi: 10.1186/s12866-018-1277-6, PMID: 30470175 PMC6251101

