# Supplementary material for: Dual suppression of Glossina pallidipes using entomopathogenic fungal-based biopesticides and sterile insect technique
Source: Front Microbiol. 2024 Dec 9;15:1472324. doi: 10.3389/fmicb.2024.1472324 (PMC11663849; doi:10.3389/fmicb.2024.1472324)
Supplement: Supplementary file 1 [file Supplementary_file_1.docx]

Supplementary Material

**Dual suppression of *Glossina pallidipes* populations using entomopathogenic fungal-based biopesticides and sterile insect technique**

Fidelis L.O. Ombura^1,2^, Adly M.M. Abd-alla^3^, Komivi S. Akutse^1,4^, Steven Runo^2^, Paul O. Mireji^5,6^, Rosemary Bateta^5^, Joseck O. Esikuri^1^, Inusa J. Ajene^1^, and Fathiya M Khamis^1,^**^β^**

^1^International Centre of Insect Physiology and Ecology (*icipe*), P.O. Box 30772-00100, Nairobi, Kenya

^2^Kenyatta University, Department of Biochemistry and Biotechnology, P.O. Box 43844 – 00100, Nairobi

^3^Insect Pest Control Laboratory, Joint FAO/IAEA Centre of Nuclear Techniques in Food and Agriculture, International Atomic Energy Agency, Wagramerstraße 5, A-1400 Vienna, Austria

^4^Unit of Environmental Sciences and Management, North-West University, Private Bag X6001, Potchefstroom 2520, South Africa

^5^Biotechnology Research Institute, Kenya Agricultural and Livestock Research Organization, P.O. Box 362-00902, Kikuyu, Kenya

^6^Centre for Geographic Medicine Research Coast, Kenya Medical Research Institute, P.O. Box 428, Kilifi, Kenya

**^β^**Author for correspondence: Fathiya M Khamis, Fax: +254 (20) 8632001, E-mail: [fkhamis@icipe.org](mailto:fkhamis@icipe.org)

# Supplementary Figures and Tables

## Supplementary Figures


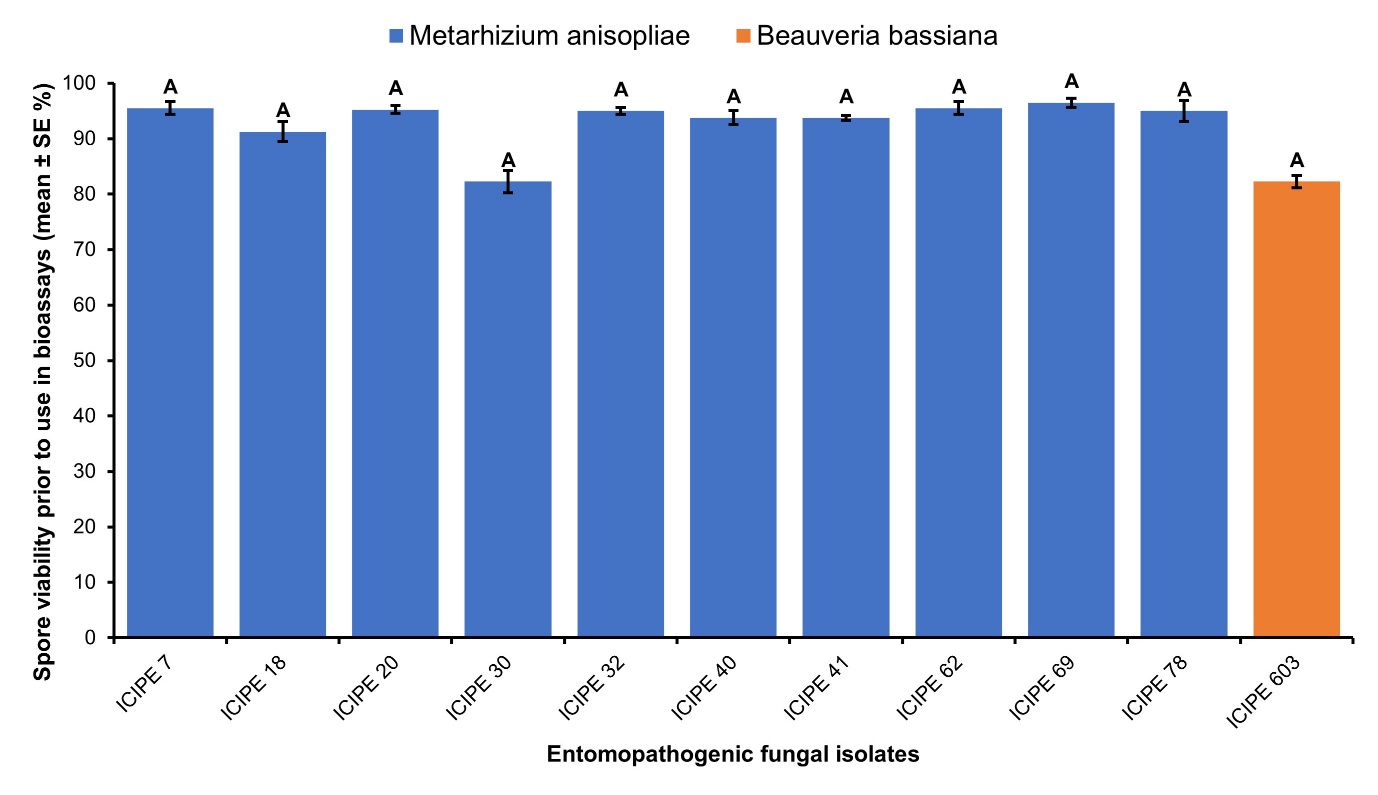


**Supplementary Figure 1**: Bar graph illustrating the mean percentage germination/spore viability rates of the 10 *M. anisopliae* strains (ICIPE 07, ICIPE 18, ICIPE 20, ICIPE 30, ICIPE 32, ICIPE 40, ICIPE 41, ICIPE 62, ICIPE 69, ICIPE 78) and the single *Beauveria bassiana* strain ICIPE 603 used in this study. Error bars indicate the standard error of the mean, and the means separation is depicted by the lowercase letters (similar lowercase letters indicate the means are not statistically significant from each other).

## Supplementary Tables

**Supplementary Table 1**: Mycosis rates of *Glossina pallidipes* cadavers infected by the entomopathogenic fungal (EPF) isolates.

|  | **Mycosis rates of EPF-challenged *Glossina pallidipes* (% ± SE)** | |
| --- | --- | --- |
| **Treatment** | **Unirradiated *G. pallidipes*** | **Irradiated *G. pallidipes*** |
| ICIPE 7 | 86.11 ± 2.76 a | 95.56 ± 0.91 A |
| ICIPE 18 | 96.30 ± 3.02 ab | 94.44 ± 1.81 A |
| ICIPE 20 | 92.93 ± 2.56 bc | 96.47 ± 1.68 A |
| ICIPE 30 | 96.67 ± 2.72 c | 100 ± 0.0 A |
| ICIPE 32 | 84.92 ± 0.65 abc | 95.16 ± 0.06 A |
| ICIPE 40 | 96.67 ± 2.72 c | 94.44 ± 0.91 A |
| ICIPE 41 | 84.13 ± 0.65 abc | 98.89 ± 0.91 A |
| ICIPE 62 | 95.31 ± 0.81 bc | 91.48 ± 1.42 A |
| ICIPE 69 | 96.54 ± 1.57 bc | 93.26 ± 0.06 A |
| ICIPE 78 | 86.11 ± 2.76 abc | 94.25 ± 0.95 A |
| ICIPE 603 | 91.67 ± 3.40 abc | 94.20 ± 1.02 A |

The values represent mean ± SE. Means within a column followed by the same letters are not significantly different (ANOVA followed by Student–Newman–Keuls (SNK) *post hoc* test, *P* < 0.05).
